# Incidental findings from cone-beam computed tomography in children and adolescents: a systematic review

**DOI:** 10.1007/s40368-025-00999-7

**Published:** 2025-01-17

**Authors:** T. Vogiatzi, S. N. Papageorgiou, N. Silikas, T. Walsh

**Affiliations:** 1 School of Medical Sciences, Division of Dentistry, The University of Manchester, Manchester, UK; 2 Clinic of Orthodontics and Pediatric Dentistry, Center for Dental Medicine, Faculty of Medicine, University of Zurich, Plattenstrasse 11, 8032 Zurich, Switzerland

**Keywords:** Dentistry, Pediatric, Radiography, Incidental finding, Pathology, Adverse effects

## Abstract

**Purpose:**

The use of cone-beam computed tomographies (CBCT) in orthodontics and pediatric dentistry is constantly increasing. The aim of this systematic review was to critically appraise and summarize evidence from clinical studies on the prevalence of incidental findings from CBCTs of children and adolescents.

**Methods:**

Systematic literature searches without restrictions were undertaken in eight databases from inception up to March 2024 for studies reporting on incidental findings from CBCT images of children and adolescents. After duplicate study selection, data extraction, and risk of bias assessment with a custom tool based on the Joanna Briggs institute’s tool for prevalence studies, qualitative (narrative) data synthesis was performed.

**Results:**

Ten studies covering a total of 1818 patients (48.5% male; average age 12.3 years) were included. The prevalence of incidental findings ranged from 0.4 to 80.3%, but high heterogeneity was seen between studies (*I*^2^ = 99%). Incidental findings were most often related to airways (63.7%), followed by bone (23.6%), teeth (19.2), spine (26.2) and temporomandibular joint (3.8%). However, several methodological issues existed with included studies (incomplete reporting of patient- or CBCT-related details, incomplete categorization and reporting on the severity of findings, small sample sizes, and research transparency issues).

**Conclusion:**

Evidence indicates that incidental findings can be often found in the CBCTs of child and adolescent patients. However, when a CBCT image is justified for children or adolescents, it should be adequately assessed for incidental findings by either a specialist oral and maxillofacial radiologist or a dentist with appropriate training and experience.

**Supplementary Information:**

The online version contains supplementary material available at 10.1007/s40368-025-00999-7.

## Introduction

A progressive increase in the use of multislice spiral computed tomography (MSCT) in diagnosis, planning, and evaluation of the outcome of dental treatment was seen in previous decades, which was associated with increased radiation exposure and cancer risk, especially of children (Brenner and Hall 2007). However, since the introduction of the cone beam computed tomography (CBCT) at the end of the twentieth century and its initial slow adoption, MSCT has mostly been replaced by CBCT. Nowadays, it is generally believed that CBCT is preferable to MSCT for the imaging of the ear and the dental arches, since it has much lower radiation dose and higher spatial resolution (Nardi et al. 2017). MSCT, on the other hand, is often preferred for imaging of the cervical spine or the head, when a higher contrast resolution is desired, despite the increased radiation (Nardi et al. 2017). As a result, currently existing clinical guidelines recommend the use of CBCT imaging for specific indications in the fields of endodontics (American Academy of Oral and Maxillofacial Radiology 2013; Patel et al. 2019), implantology (Rios et al. 2017), periodontology (Kim and Bassir 2017), oral and maxillofacial surgery (Hayashi et al. 2018; Assouline et al. 2021), orthodontics (American Academy of Oral and Maxillofacial Radiology 2013; Christell et al. 2018; Sosars et al. 2020), paediatric dentistry (Kühnisch et al. 2020), cleft lip and palate (Kapila and Nervina 2015), and temporomandibular joint disorders (Kapila and Nervina 2015; Kim and Bassir 2017; Almashraqi et al. 2024).

Depending on the chosen field of view (FOV), small tissue like single teeth up to the full dentomaxillofacial complex or even the entire human head may be depicted. On the other hand, CBCT images with a large FOV (height > 15 cm) might be used to visualize whole areas like the paranasal sinues, the whole upper airway, intracranial structures or structures of the cranial base and might include a wide range of depicted tissues (teeth, bone, soft tissues, air cavities, etc.) (Kühnisch et al. 2020). Inadvertently, there exists a possibility that such radiographic images might reveal findings that are not related to the initial reason for which the diagnostic imaging was prescribed. Indeed, such incidental findings have been reported in many imaging modalities of several body areas including the chest (Waterbrook et al. 2018), spine (Ramadorai et al. 2014), or brain (Eskandary et al. 2005). A recent umbrella review of systematic reviews on incidental findings reported that there is large variability across the prevalence and severity of findings from biomedical imaging modalities, including positron emission tomography, computerized tomography, and magnetic resonance imaging of various body parts (breast, colon, heart, parotid, spine, thorax, thyroid, etc.) (O’Sullivan et al. 2018).

However, incidental findings from CBCT imaging studies on children have rarely been assessed. Current guidelines from the European Academy of Paediatric Dentistry (EAPD) indicate that very strict indications for CBCTs on children exist and its use should be limited to the few clinical situations where two-dimensional imaging modalities fall short in terms of diagnostic efficacy. Additionally, the smallest FOV, the largest voxel, ultra-low dose settings, and a thyroid shield should be used, whenever possible without compromising the diagnostic needs of the CBCT (Kühnisch et al. 2020). The same guidance document clearly states that incidental findings of clinical significance are rare in CBCTs of children, and therefore, screening for their possible presence cannot justify their prescription.

An older systematic review on CBCT incidental findings of the head and neck region among various patients found that these are detected relatively frequently, and considerable variation is evident in their frequency and nature (Edwards et al. 2013). Another more recent systematic review on CBCT incidental findings across a wide variety of patients reported that these are frequently observed, but not all of them require immediate medical attention (Dief et al. 2019). The third, and most recent, systematic review of CBCT incidental findings of the maxillofacial region reported prevalences ranging from 0.3 to 71.1% (Khalifa and Felemban 2021), with varying severity and without clear consensus on their recommended management. However, the authors of these reviews emphasized that, when CBCT images are acquired, the entirety of this image should be reviewed by a trained specialist for any incidental finding, regardless of FOV or region of interest (ROI). Existing systematic reviews on the subject have included (i) studies published up to 2020, (ii) only studies in English, and (iii) patients of any age without reporting separate findings for underage and adult patients.

Therefore, the aim of the present review was to determine the prevalence and nature of incidental findings in CBCTs of children and adolescents, as they often receive such radiographs during the primary and mixed dentition phase for diagnostic or treatment-related reasons.

## Materials and methods

### Protocol and registration

This review’s protocol was made a priori, registered in PROSPERO, all deviations were transparently reported (Supplement), while the conduct and reporting of this review was guided by the appropriate chapter of the Joanna Briggs Institute (JBI) Manual for Evidence Synthesis (Gibson et al. 2018).

### Eligibility criteria

The Participants—Exposure—Comparison—Outcome—Study design (PECOS) framework was used to formulate this review’s research question: What incidental findings can be found in CBCTs of children and adolescents from observational studies (Supplementary Table S1). Studies with healthy patients up to 18 years of age, of any sex or ethnicity with any kind of anamnestic or clinical justification deemed in need to receive a CBCT for diagnostic imaging reasons were eligible for inclusion. If studies reported on mixed samples of children and adults, we contacted the authors to request data of the non-adult patients.

The condition of interest was any incidental finding from CBCT images. Any clinical setting was included to increase the applicability/generalizability of the results. Other important outcomes were the prevalence of any specific clinical outcomes. We included studies of CBCT of the head/neck region for diagnostic reasons and excluded other imaging modalities, radiography used for radiotherapy of cancers, and radiographs of other body regions. We included studies of assessments of CBCT images done either longitudinally or cross-sectionally (and both in a prospective and retrospective manner). Animal studies, case series/reports, and non-clinical studies (i.e. cadaver studies) were excluded.

### Search strategy

We searched the following electronic general, open access, regional and grey literature bibliographic databases: MEDLINE (searched via PubMed), Embase, The Cochrane Library (CDSR, CENTRAL, and DARE), Virtual Health Library (including Bibliography Brazilian Dentistry and LILACS), Scopus, and ISI Web of Knowledge (Supplementary Table S2) up to March 2024. Additionally, Directory of Open Access Journals (DOAJ), Digital Dissertations (searched via UMI Proquest), metaRegister of Controlled Trials, WHO trials search portal, and Google Scholar were searched manually. No restrictions were placed regarding publication year/language/status and no search filters were applied other than studies on humans, where available. Hand searching was also performed from the reference/citation lists of the full text articles that could be eligible for inclusion and relevant systematic reviews.

### Study selection and data extraction

Two review authors (TV and SNP) screened the titles and/or abstracts of studies retrieved from the searches and those from additional sources (hand searching, reference/citation lists) to identify articles that potentially met the inclusion criteria. The full text of these potentially eligible studies, as well as of those abstracts which did not provide sufficient information to allow decision-making as regards inclusion or exclusion, were retrieved and assessed by one review author (TV), while a second checked the decisions (SNP). Any differences between the two reviewers were discussed and settled by consensus and, if needed, by consulting a third author (NS or TW).

Data collection from the identified reports was conducted using pre-defined forms that were finalized and piloted by two authors (TV and SNP) prior to the completion of the literature searches. Data extraction was undertaken independently and in duplicate including study design, setting/location, country of origin, number of patients, sex, age, ethnicity, reason to acquire the CBCT image, CBCT technical details, CBCT assessor, and reported findings. Discrepancies were resolved as above. Where possible, authors were contacted to obtain the missing data.

### Risk of bias assessment

The risk of bias within included studies was assessed using JBI’ Critical Appraisal Instrument for Studies Reporting Prevalence Data (Munn et al. 2015) after appropriate modifications/additions to tailor the use of the tool to the systematic review (Supplementary Table S3) pertinent to the focus of the review and after checking previous systematic reviews (Edwards et al. 2013; Gibson et al. 2018; O’Sullivan et al. 2018; Dief et al. 2019; Munn et al.^2020^; Khalifa and Felemban 2021; Sunny et al. 2022).

### Data synthesis

Initially, quantitative data synthesis (meta-analysis) of prevalence was planned, but was ultimately not performed, due to the existence of scarce and heterogeneous studies. Instead, evidence synthesis was limited to reporting the prevalence of each incidental finding reported from included studies, using the number of patients with a particular incidental finding divided by the total number of patients assessed in the study (with and without any incidental findings) and Freeman-Tukey double arcsine transformation to calculate the 95% confidence intervals of single studies (Lin and Xu 2020). Additional analyses (subgroup analyses, meta-regression analyses, sensitivity analyses) were planned but could ultimately likewise not be conducted. The review’s dataset was made openly available through Zenodo (Vogiatzi et al. 2024).

### Certainty of evidence

The certainty of available evidence was not formally addressed, since no formal guidance exists for systematic review of prevalence studies. Interpretation of the studies’ results was performed in accordance with their risk of bias.

## Results

### Study selection

**Fig. 1:**
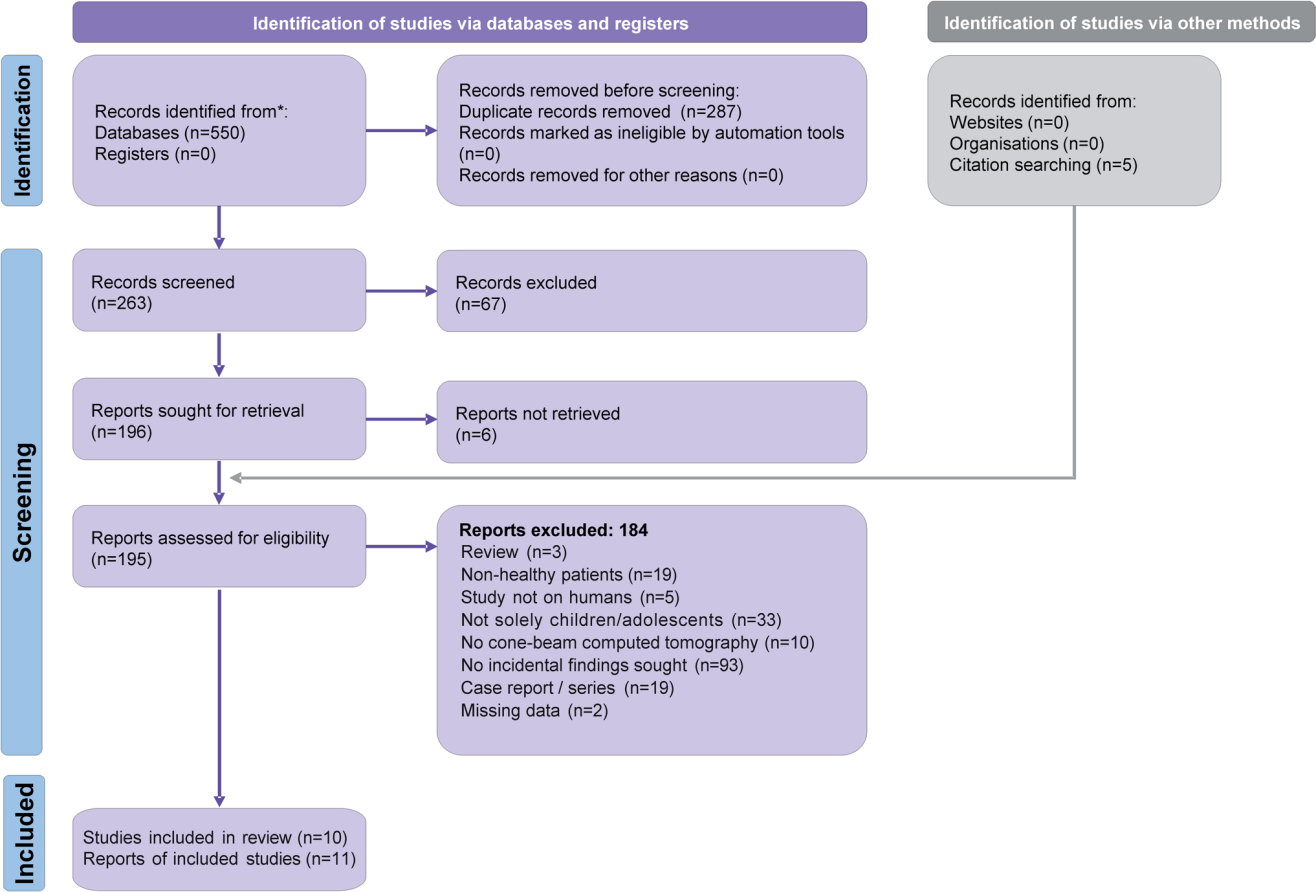
PRISMA flow diagram for the identification/selection of eligible studies

The electronic records search yielded a total of 550 records with a search of additional resources (hand-searching) yielding five records. Removal of duplicates resulted in 263 unique records (Supplementary Table S4). After removing obviously non-relevant records from title/abstract, 195 reports were ultimately checked against the eligibility criteria (Fig. 1). Initially, six papers reporting on six unique studies fulfilled the review’s eligibility criteria and were included in the review. Furthermore, another 25 potentially eligible studies reporting on mixed child/adult study samples were identified. The study authors were contacted to request data on solely children (Supplementary Table S5). Finally, raw datasets were received for four of these studies, which were included—to a total of 11 published reports pertaining to 10 unique studies (Cobb 2013; Bayrakdar et al. 2014; Cantekin and Şekerci 2014; Dogramaci et al. 2014; Etemad et al. 2023; Geist et al. 2014; Lopes 2016; Togan et al. 2016; Alsufyani 2017; Lopes et al. 2017).

### Study characteristics

Nine studies were published as journal articles, one as Master thesis, and one as both Master thesis and journal article. All studies were in English, apart from one thesis that was published in Portuguese. All studies were cross-sectional in nature (Table 1), their majority (9/10; 90%) were performed from university clinics, and originated from seven countries (Brazil, Canada, Great Britain, Italy, Switzerland, Turkey, and United States of America). The median study sample size was 143.5 patients (interquartile range 48 to 267 patients) with equal distribution of gender (48.5% male; 492/1014, from the 6 studies reporting on gender). The average patient age was 12.3 years (from the 8 studies reporting on age), while ages ranged from 3 to 18 years. Patient ethnicity was infrequently reported, while CBCTs were taken for a variety of reasons, including impactions, orthodontics, pathology, supernumerary teeth, temporomandibular joint assessment, cysts, surgery, 3rd molars, implant placement, prosthetic rehabilitation, and orthognathic surgery.

**Table 1:** Characteristics of included studies; patient characteristics

Study	Data	Design; setting; country*	Children (M/F)	Mean age (range)	Ethnicity	CBCT reason
Alsufyani (2017)	Raw	CS; Uni; CAN	27 (11/16)	13.6 (8.0 to 18.0)	NR	Various (implants, ortho, pathology, TMJ)
Bayrakdar (2014)	Paper	CS; Uni; TUR	178 (NR)	NR (8.0 to 18.0)	NR	NR
Cantekin (2014)	Paper	CS; Uni; TUR	275 (139/126)	10.5 (3.0 to 15.0)	NR	Diagnosis and Tx planning (impactions, ortho, pathology, supernumerary, TMJ)
Cobb (2013)	Paper	CS; MilClin; USA	267 (NR)	13.7 (NR to 18.0)	NR	Various (implants, ortho, pathology, orthognathic Tx, prostho, surgery, TMJ)
Dogramaci (2014)	Raw	CS; Uni; GBR	109 (NR)	13.6 (7.0 to 18.0)	NR	Maxillary impacted canines
Etemad (2023)	Paper	CS; Uni; USA	250 (NR)	NR (13.0 to 18.0)	NR	Ortho (impaction, clefts, third molar)
Geist (2014)	Paper	CS; Uni; USA	576 (271/305)	12.2 (5.0 to 17.0)	Various	Ortho diagnosis and Tx planning
Giaccaglia (2022)	Paper	CS; Uni; ITA	61 (32/29)	11.0 (5.0 to 14.0)	NR	‘Ortho purposes’
Lopes (2016, 2017)	Raw	CS; Uni; BRA	27 (14/13)	13.1 (8.0 to 17.0)	NR	Various (Cyst, implant, ortho, supernumerary, surgery, 3rd molar)
Togan (2016)	Raw	CS; Uni; CHE	48 (25/23)	13.8 (6.7 to 17.8)	NR	NR

CBCT, cone beam computed tomography; CS, cross-sectional; MilClin, military clinic; ortho, orthodontic(s); NR, not reported; TMJ, temporomandibular joint; Tx, treatment; Uni, university

^*^Country given with the ISO alpha-3 code

The included studies were diverse in their design and conduct, precluding a quantitative synthesis. The CBCTs were performed with different appliances and different settings, with varying fields of view, and voxel size (0.16 to 0.40 mm) (Table 2). The CBCT’s areas of interest likewise ranged from parts of the jaws (maxilla, mandible, or both) to specific areas of the cervical area or the nasal/paranasal sinuses. The acquired CBCT images were assessed by specialized oral/maxillofacial radiologists (50%; 5/10 studies), vaguely-phrased ‘radiologists’ (20%; 2/10 studies), ‘dental specialists’ (10%; 1/10 study), maxillofacial surgeons (10%; 1/10 study), or was not reported at all (10%; 1/10 study). The acquired CBCTs were checked for either specific incidental findings of some structures like the spine, mental foramen, and the maxillary sinus (60%; 6/10 studies) or underwent complete assessments of the covered field to identify possible incidental findings pertaining to all imaged structures (40%; 4/10 studies). Even though all studies reported in their articles (or provided after contacting them) the number of patients with CBCT incidental findings, two (20%) did not report the initial number of CBCTs that were assessed (of patients with or without incidental findings).

**Table 2:** Characteristics of included studies; radiographic assessment details

Study	CBCT details (FOV; site)	CBCT evaluation	Findings reported
Alsufyani (2017)	iCAT Classic/Next Generation; varying FOV and resolution; cervical spine and clivus	3 × oral/maxillofacial radiologist	Spine/clivus findings (ossicle, fusion, scoliosis, subluxation, foveolar, ponticulus posticus, etc.)
Bayrakdar (2014)	NewTom 3G; 110 kv; 15 mAs; 0.16 mm voxel; 36”	2 × radiologist	Ponticulus posticus
Cantekin (2014)	NewTom 5G; 110 kv; 15 mA; 0.16–0.30 mm voxel; 36”	2 × ‘dental specialist’	Accessory mental foramen
Cobb (2013)	(A) iCAT Classic; 13 × 16 cm; 120 kV; 5 mA; 0.3 mm voxel; 20”; (B) iCAT Platinum; 17 × 23 cm; 120 kV; 5 mA; 0.3 mm voxel; 17.8”	1 × oral/maxillofacial radiologist	Complete assessment (airway, dental, nasal cavity, osseous structures, paranasal sinus, TMJ)
Dogramaci (2014)	3D Accuitomo 80; 40 × 40 cm or 60 × 60 cm FOV; 70–90 kV; 3.0–4.0 mA; 17.5”	Oral/maxillofacial radiologists	Complete assessment (follicle, sinus mucosal thickening, dilacerations, cyst, pulp stone, caries, etc.)
Etemad (2023)	Accuitomo; 17 × 20 cm FOV; 90 kV; 10.0 mA; 17.5”	1 × dental student and 2 × oral/maxillofacial radiologist	Complete assessment [(i) sino-nasal, (ii) dentoalveolar, (iii) naso-oropharyngeal airway, (iv) temporomandibular joint, (v) neck, (vi) calcifications, and (vii) miscellaneous findings]
Geist (2014)	iCAT 17–19; ≥ 16 × 18 cm; 120 kV; 5 mA; 0.3 mm voxel; 8.9”	1 × oral/maxillofacial radiologist	Ponticulus posticus; Ponticulus lateralis
Giaccaglia (2022)	NR; paranasal sinus and nasal septum	NR	Septum anatomical variations; sinus mucosal thickening; sinus cyst or polypoid formation; sinus underdevelopment
Lopes (2016, 2017)	iCAT Classic; 6–13 cm FOV; 0.2–0.4 mm voxel; one or both jaws	2 × radiologists	Complete assessment (airway, bone, calcifications, jaw lesions, teeth, TMJ)
Togan (2016)	KaVo 3D exam; 90–120 kV; 3–8 mA; 0.4 mm voxel; 8.5”; sinus and/or cervical area	Maxillofacial surgeon	Cyst; sialoliths; paranasal sinus findings; stylohyoid ligament calcification

CBCT, cone-beam computed tomography; CS, cross-sectional; FOV, field of view; MilClin, military clinic; ortho, orthodontic(s); prostho, prosthodontics; TMJ, temporomandibular joint; Tx, treatment; Uni, university

^*^Country given with the ISO alpha-3 code

### Risk of bias/issues with the internal validity of included studies

The assessment of methodological characteristics and internal validity factors of included studies can be seen in Supplementary Table S6. Most problematic domains were non-reported or incomplete reporting of the CBCT’s technical settings (70% of the cases), non-reported patient eligibility criteria (60% of the cases), non-complete assessment/reporting of all possible incidental findings (40% of the cases), limited sample sizes leading to potentially small-study effects (40% of the cases), lack of differentiation between minor-severe incidental findings (40% of the cases) and the lack of reason provided for acquiring a CBCT (20% of the cases). Additionally, important study information was not transparently explained, including blinding of the CBCT assessors (80% of the cases), lack of consistent conditions for all acquired CBCTs (80% of the cases), lack of appropriate/controlled conditions for the assessment of included CBCTs (70% of the cases), duplicate assessment of each CBCT (60% of the cases), and lack of measures put in place to minimize selection bias (60% of the cases).

### Results of individual studies

**Fig. 2:**
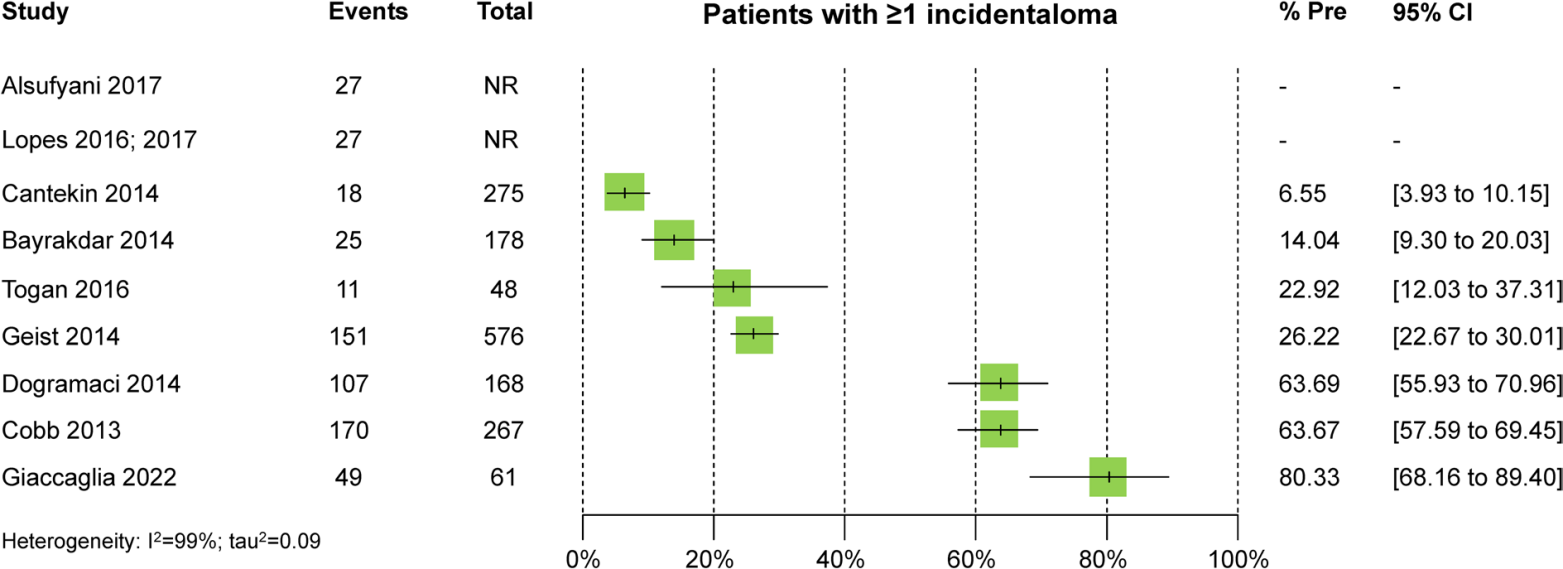
Forest plot of % prevalence of patients with at least one incidental finding. CI, confidence interval; NR, not reported; Pre, prevalence

Results of the included studies were reported inconsistently in the identified reports. Lopes (2016) and Alsufyani et al. (2017) gave the total sample of CBCTs that were assessed (with or without incidental findings). Cobb (2013) reported data only on incidental finding level, including multiple instances of the same incidental finding within a patient (but not on patient level), and highlighted which incidental findings were most often found among the total number of incidental findings (but not among the total number of assessed CBCTs). Among the studies providing this data, incidental findings were identified in 6.5–80.1% of assessed patients, depending on each study’s characteristics, design, and scope (Fig. 2).

From studies reporting only on patients with incidental findings, the percentages of each identified incidental finding are summarized for each study in Supplementary Tables S7–S13. Alsufyani et al. (2017) assessed incidental findings of the spine/clivus and reported (i) congenital fusions, misalignment, and scoliosis of the cervical vertebrae, normal variations (grooves, foveolas, and ponticuli postici) and degenerative joint disease. Incidental findings of ponticuli postici were similarly reported from Bayrakdar et al. (2014) and Geist et al. (2014), while Cantekin and Şekerciet (2014) reported on the prevalence of an accessory mental foramen. Togan et al. (2016) reported mostly on sinus findings, followed by submandibular sialolithiasis, calcification of the stylus-hyoid complex, and tonsiloliths (Supplementary Table S11). Lopes (2016) reported on multiple findings pertaining mostly to the teeth (impactions, rotations, dilacerations), followed by the temporomandibular joint (osteophytes and condylar flattenings), the airways (sinus mucosal thickenings, cysts, turbinate hypertrophy, and nasal septum deviations), soft tissue calcifications (mostly of the stylus–hyoid complex), and bone alterations (Supplementary Tables S9–S10). Comprehensive assessments of CBCT incidental findings covering diverse tissues/areas (teeth, bones, TMJ, airways) performed only by Cobb (2013), Dogramaci et al. (2014), and Etemad et al. (2023). Cobb and Etemad reported that the most often found incidental findings were located at the area of the sino-nasal and naso-oropharyngeal airway (adenoid and tonsillar hypertrophy, concha bullosa and septum deviation, mucosal thickenings, pneumatizations, and pseudocysts). According to Cobb, the osseous structures (high-density areas and vertebrae defects) and the dentition (missing teeth, impactions, lesions, retentions, and supernumeraries) follow the airway findings, whereas according to Etemad, the dentoalveolar findings are the second more frequently found incidental findings, followed by the calcifications-findings (mainly the calcification of the stylohyoid ligament). The calcification of the stylohyoid ligament was also mentioned in the study of Cobb, with a prevalence of 10.03%. On the other hand, Dogramaci reported that the majority of incidental findings concerned the “teeth” category (enlarged follicle, root dilaceration, abnormal morphology) followed by the airway findings and the bone lesions. However, these teeth subcategories examined by Dogramaci, where not included in the finding tables of the other two studies.

From studies actually reporting both number of patients with incidental findings and the total number of assessed CBCTs, the absolute prevalence of each identified incidental finding is presented in Table 3. Incidental findings of any kind at the airways and the nasal/paranasal cavities were found in more than half of assessed patients (prevalence 49.8 to 63.7%). Among the most often found incidental findings were mucosal thickening of the sinuses, presence of Onodi cells, nasal septum deviations, and concha bullosa cases. Incidental findings of the temporomandibular joint were comparatively rare, with a prevalence of about 3.7%. Dental incidental findings of any kind were identified at about every 5th patient (19.1–19.2%), with the most prevalent incidental findings being enlarged dental follicles, root dilacerations, abnormal tooth morphology, tooth impactions, pulp calcifications, and caries. Incidental findings pertaining to bone structures were found in about 23.6% of all patients, with thinning of the cortical bone, ossicles, and bone dehiscences being most often observed. Cysts were identified in 1.2 to 4.8% of assessed patients, accessory mental foramen in 6.5% of the cases, and ponticuli posticus/lateralis in 6.1–26.2% of the cases.

**Table 3:** Prevalence of incidental findings (patient level) from included studies; number of patients with incidental findings by total study sample

Category	Finding	Tx need	Absolute*	%	95% CI
Airway	Any airway finding	–	170/267^3^	63.67	57.59–69.45
		–	112/250^7^	44.80	38.53–51.19
	Any nasal cavity finding	–	133/267^3^	49.81	43.66–55.97
	Any naso-oropharyngeal finding	–	50/250^7^	20.00	15.22–25.50
	Any paranasal sinus finding	–	149/267^3^	55.81	49.62–61.86
	Any frontal sinus finding	–	98/250^7^	39.20	33.11–45.55
	Accessory ostium	–	19/250^7^	7.60	4.64–11.61
	Concha bullosa	–	3/168^4^	1.79	0.37–5.13
			5/61^6^	8.20	2.72–18.10
		–	44/250^7^	17.60	13.09–22.90
	Enlarged adenoids	–	151/250^7^	60.40	54.04–66.51
	Enlarged tonsils	–	47/250^7^	18.80	14.15–24.20
	Haller cells	–	1/61^6^	1.64	0.04–8.80
		–	11/250^7^	4.40	2.22–7.74
	Onodi cells	–	14/61^6^	22.95	13.15–35.50
	Ostia blockage	–	3/250^7^	1.20	0.25–3.47
	Mastoid cells over TMJ	–	17/250^7^	6.80	4.01–10.66
	Mucous retention cysts	Yes	40/250^7^	16.00	11.68–21.14
	Nasal floor perforation	–	4/168^4^	2.38	0.65–5.98
	Nasal septum deviation	–	14/168^4^	8.33	4.63–13.59
		–	49/61^6^	80.33	68.16–89.40
		–	135/250^7^	54.00	47.61–60.30
	Pansinusitis	Yes	4/250^7^	1.60	0.44–4.05
	Sinus mucosal thickening; maxillary sinus	–	43/168^4^	25.60	19.18–32.89
		–	43/61^6^	70.49	57.43–81.48
		–	119/250^7^	47.60	41.27–53.99
	Sinus mucosal thickening; maxillary sinus (bilat.)	–	13/168^4^	7.74	4.18–12.87
	Sinus mucosal thickening; ethmoid sinus	–	18/61^6^	29.51	18.52–42.57
	Sinus mucosal thickening; sphenoid sinus	–	21/61^6^	34.43	22.73–47.69
	Sinus mucosal thickening; ethmoid and sphenoid sinus	–	5/250^7^	2.00	0.65–4.61
	Sinus mucosal thickening; frontal sinus	–	10/61^6^	16.39	8.15–28.09
	Sinus underdevelopment; frontal sinus	–	20/61^6^	32.79	21.31–46.00
	Sinus underdevelopment; ethmoid sinus	–	1/61^6^	1.64	0.04–8.80
	Sinus underdevelopment; sphenoid sinus	–	2/61^6^	3.28	0.40–11.35
	Sinus underdevelopment; maxillary sinus	–	1/61^6^	1.64	0.04–8.80
	Sinus polyp; maxillary sinus	–	1/168^4^	0.60	0.02–3.27
			1/61^6^	1.64	0.04–8.80
	Sinus polyp; ethmoid sinus	–	2/61^6^	3.28	0.40–11.35
	Tonsilolith	–	17/250^7^	6.80	4.01–10.66
	Vascular calcification	–	2/250^7^	0.80	0.10–2.86
TMJ	Any TMJ finding	–	10/267^3^	3.75	1.81–6.78
		–	1/250^7^	0.40	0.01–2.21
	Osteoarthritis	–	3/250^7^	1.20	0.25–3.47
Teeth	Any dental finding	–	51/267^3^	19.10	14.57–24.34
		–	48/250^7^	19.20	14.51–24.64
	Ankylosis	Yes	3/168^4^	1.79	0.37–5.13
	Abnormal morphology	–	27/168^4^	16.07	10.87–22.51
	Caries	Yes	11/168^4^	6.55	3.31–11.41
	Dens invaginatus	–	4/168^4^	2.38	0.65–5.98
	Ectopic tooth position	–	3/250^7^	1.20	0.25–3.47
	Enamel pearl	–	1/168^4^	0.60	0.02–3.27
	Enlarged follicle	–	61/168^4^	36.31	29.04–44.07
	Microdontia	–	2/250^7^	0.80	0.10–2.86
	Odontoma	Yes	3/168^4^	1.79	0.37–5.13
		Yes	2/250^7^	0.80	0.10–2.86
	Pericoronitis	Yes	1/250^7^	0.40	0.01–2.21
	Periodontal ligament widening	–	7/168^4^	4.17	1.69–8.40
	Pulp calcification	–	11/168^4^	6.55	3.31–11.41
	Retained root	Yes	7/168^4^	4.17	1.69–8.40
	Root dilaceration	–	57/168^4^	33.93	26.81–41.62
		–	1/250^7^	0.40	0.01–2.21
	Root fracture	Yes	1/168^4^	0.60	0.02–3.27
	Root resorption	Yes	9/168^4^	5.36	2.48–9.93
		Yes	2/250^7^	0.80	0.10–2.86
	Short root	–	8/168^4^	4.76	2.08–9.17
	Supernumerary teeth	Yes	3/168^4^	1.79	0.37–5.13
		Yes	11/250^7^	4.40	2.22–7.74
	Third molar finding	–	108/250^7^	43.20	36.97–49.59
	Tooth agenesis	Yes	1/168^4^	0.60	0.02–3.27
		Yes	29/250^7^	11.60	7.91–16.23
	Tooth impaction	Yes	21/168^4^	12.50	7.91–18.47
	Tooth transposition	–	3/250^7^	1.20	0.25–3.47
Bone	Any osseous structure finding	–	63/267^3^	23.60	18.63–29.15
	Bone dehiscence	–	1/168^4^	0.60	0.02–3.27
	Bone sclerosis	–	1/168^4^	0.60	0.02–3.27
		–	2/250^7^	0.80	0.10–2.86
	Ossicle	–	5/168^4^	2.98	0.97–6.81
	Thinning of cortical bone	–	27/168^4^	16.07	10.87–22.51
Jaw lesions	Cyst	Yes	2/168^4^	1.19	0.14–4.23
	Dentigerous cyst	Yes	8/168^4^	4.76	2.08–9.17
		Yes	3/250^7^	1.20	0.25–3.47
Mandible	Accessory mental foramen	–	18/275^2^	6.55	3.93–10.15
	Torus mandibularis	–	1/250^7^	0.40	0.01–2.21
Spine	Any neck finding	–	1/250^7^	0.40	0.01–2.21
	Ponticulus posticus	–	25/178^1^	14.04	9.30–20.03
		–	151/576^5^	26.22	22.67–30.01
	Ponticulus lateralis	–	35/576^5^	6.08	4.27–8.35
	Cervical osteoarthritis	–	2/250^7^	0.80	0.10–2.86
Other	Any other finding	–	133/267^3^	49.81	43.66–55.97
		–	3/250^7^	1.20	0.25–3.47
	Segmental maxillary odontodysplasia	–	1/250^7^	0.40	0.01–2.21
	Any calcification	–	36/250^7^	14.40	10.29–19.37
	Stylohyoid ligament calcification	–	202/250^7^	80.80	75.36–85.49
	Petroclinoid calcification	–	1/250^7^	0.40	0.01–2.21
	Thyroid cartilage calcification	–	1/250^7^	0.40	0.01–2.21
	Falx cerebri calcification	–	1/250^7^	0.40	0.01–2.21

^*^Study notations used: ^1^Bayrakdar (2014); ^2^Cantekin (2014); ^3^Cobb (2013); ^4^Dogramaci (2014); ^5^Geist (2014); ^6^Giaccaglia (2022); ^7^Etamad (2023)

TMJ, temporomandibular joint; Tx, treatment

## Discussion

This systematic review summarizes evidence from existing observational studies on incidental findings from CBCTs from children and adolescents. Data from the ten included studies covering a total of 1818 patients indicated that incidental findings are often seen (6.6–80.3%) and pertain to findings related to the airways, the alveolar bone, the teeth, or the temporomandibular joint. However, not all incidental findings are of equal significance and do not always warrant intervention. A previous systematic review on incidental findings from both underage and adult patients (Dief et al. 2019) reported similar overall prevalences (24.6–94.3%) and agreed that even though not all of them require immediate medical attention, they should nonetheless be fully evaluated.

The classification of the severity of the incidental findings was examined in 4 of the included studies (4/10). Dogramaci et al. (2014) reported that incidental findings in small-volume CBCT scans focused on impacted maxillary canine teeth required immediate action in a small minority of cases (0.4%), 28.5% required follow-up and the remainder 71.1% were classified as either low-grade or anatomic variant. Lopes (2016) who used 3 different FOVs, reported that 43.46% of incidental findings did not need treatment and/or referral, 28.97% required follow-up and 27.57% needed treatment and/or referral to another professional. Cobb (2013) reported an incidence of 17% and 27% for the severe and moderate findings respectively for the group of children/adolescents using the medium and large FOV. Etemad et al. (2023) who examined CBCTs with large FOV, found that the percentage of incidental findings that were assigned a rating of mild was 27%, moderate was 72%, and severe was 1%.

However, it was impossible to carry out a full direct comparison between the previously mentioned publications and report on the clinical importance and significance of incidental findings because of the high variability and differences in the categories used to classify findings, the variety of exposure settings and field of view, the inconsistency of the assessment by a variety of dental professionals, the difference in reporting at the total patients sample level or at incidental finding patients level, as well as the obvious difficulties in reaching a final diagnosis based solely on CBCT.

It must be noted here that great inconsistency existed among both the reported findings and the severity assessment of findings from authors of included studies. For example, Cobb (2013) reported adenoid and/or tonsillar hypertrophy at 15.6% and classified this as severe/moderate (even though these are often simply variations in growth and development), while Etemad et al. (2023) reported overall similar percentage of incidental findings in the naso-oropharyngeal area (20%) but classified this as moderate—resulting in higher incidence of moderate findings as other studies (Cobb 2013; Doğramacı et al. 2014; Lopes et al.^2017^). Additionally, the percentage of the severe incidental findings, classified as needing immediate action or referral, increased accordingly to the size of the FOV (0.4% for the small FOV, 17–27% for the medium and large FOV). Therefore, standardization of this process (including appropriate FOV, severity of findings, whether specialist maxillofacial radiologists are needed for proper diagnosis, and whether blinding should be employed and in which extent), would be helpful towards comparatibility of future studies.

It is important here to note that research on this subject is wrought with difficulties due to the nature of the research question. For one, a large number of CBCT radiographs on children need to be available for diagnostic reasons, which at this age are usually requested by oral/maxillofacial radiologists, pediatric dentists, or orthodontists (Ismayılov and Özgür 2023) and might most probably be related to clarifications of tooth agenesis (Walliczek-Dworschak et al. 2017; De Grauwe et al. 2019; Hajem et al. 2020; Ismayılov and Özgür 2023), the existence of supernumerary teeth (Ismayılov and Özgür 2023), dental anomaly (De Grauwe et al. 2019), tooth impaction (De Grauwe et al. 2019; Hajem et al. 2020), root resorption of adjacent teeth (Hajem et al. 2020), root fracture and condylar shape/volume (De Grauwe et al. 2019), and facial/dental trauma (Walliczek-Dworschak et al. 2017), pre-surgical diagnosis/treatment planning of cleft lip and palate patients and orthognathic surgery (De Grauwe et al. 2019). According to the original indication for CBCT imaging, different areas of the maxillofacial complex might be imaged or different fields-of-view might be chosen, with the largest portion of CBCT being focused on the maxilla [35.0% (Ismayılov and Özgür 2023)] and using a small field-of view (≤ 10 cm) [53.35% (Ismayılov and Özgür 2023)]. However, regardless of the reported indications from published studies, lists of specific indications for CBCTs of underage patients exist both from the SEDENTEXCT project of the European Commission (2012) and the DIMITRA (dentomaxillofacial paediatric imaging investigation towards low-dose radiation) project (Oenning et al. 2018).

Subsequently, even if a sufficient number of CBCTs of underage patients are available, they need to be thoroughly examined by assessor with the appropriate level of knowledge and expertise. Among the included studies, the acquired CBCTs were inconsistently assessed by a variety of dental professionals, including oral maxillofacial radiologists, maxillofacial surgeons, or assessors of unspecified qualifications. The most prevalent opinion is that CBCT scans should be read by adequately trained dental practitioners or, preferably, by a specialist oral and maxillofacial radiologist (Horner et al. 2009). In some countries like Sweden, there is even strict regulation that a specialist in oral/maxillofacial radiology must confirm the justification for a CBCT and oversee its examination (Hajem et al. 2020). In few European countries like Norway, Sweden, UK, Finland and Turkey, the dental and maxillofacial radiology is recognized as a registered specialty with formal training curriculum (Brown et al. 2014). However, the general notion is that non-radiologist dentists should not be excluded from performing or interpreting CBCT imaging, as long as they have appropriate and documented training and experience (Scarfe et al. 2006).

Another important issue regarding the use of CBCT imaging is that recommended indications are not absolute and critical appraisal of each case is warranted. For example, even though canine impactions is one of the most often cited reasons for CBCT imaging (De Grauwe et al. 2019; Hajem et al. 2020), evidence on their diagnostic value is conflicting. There are several studies that indicate that CBCT imaging does not consistently improve diagnostic accuracy or treatment planning of canine impaction cases (Alqerban et al. 2014; Christell et al. 2018) and that small volume CBCT may be justified in terms of added benefit only when root resorption of adjacent teeth is suspected, when the canine apex is not clearly discernible in the panoramic X-ray, or when the impacted canine has a large alpha or beta angle (Alqerban et al. 2014; Christell et al. 2018; Stoustrup et al. 2024). The evidence is similarly not unanimous about the use of CBCT for the surgical treatment planning of third molars and should only be used for selected cases for which the surgeon has specific clinical question to be answered (such as the proximity of the third molar to the mandibular canal) (Matzen et al. 2013; Araujo et al. 2019; Matzen et al. 2020)—which was also adopted by the European Commission made the European Academy of DentoMaxilloFacial Radiology (EADMFR) (Matzen and Berkhout 2019). Therefore, it is important that CBCT is not routinely in clinical dental practice, but its use is guided by appropriate appraisal of each case and a cost–benefit analysis (Matzen and Berkhout 2019).

The present review has several limitations, which affect our certainty about what clinical recommendation can be currently made. Only a limited number of studies with mostly small sample sizes was found and could be included, with 40% of them assessing less than 100 CBCTs. Many identified studies were excluded since they assessed mixed samples of both underage and adult patients, did not report results separately for each age group, and did not respond to our communication attempts (Supplementary Table S5). Additionally, only a few studies performed a complete assessment of all possible incidental findings of the radiographed areas and mostly reported on a handful of arbitrarily selected findings. Furthermore, acquired CBCTs were not always assessed by a specialist oral and maxillofacial radiologist or a dentist with adequate training and experience. Furthermore, the conditions under which the acquired CBCT images were read was disregarded in most studies, even though evidence indicates that room lighting and image display device have been shown to affect reported findings (Kallio-Pulkkinen et al. 2015; Baltacıoĝlu et al. 2016; Cruz et al. 2018; Harvey and Patel 2020). Moreover, the studies included in this systematic review assessed children’s populations with an age range from 5 to 18 years old. This is a very wide range, since it includes different developmental phases, in which anatomical maturation as well as variability in maturation take place. Assessors of pediatric CBCTs need to be aware of these normal developmental changes so as not to misinterpret normal changes or anatomical variations for pathology, underlining once again the necessity of the implementation of specialists in the diagnosis and interpretation of the CBCTs. Finally, existing data originate from developed countries (Brazil, Canada, Great Britain, Italy, Switzerland, Turkey, and United States of America) and might not be easily generalizable to other developing countries.

## Conclusion

Existing evidence indicates that incidental findings are often seen in the cone-beam computed tomographies of children and adolescents, but there is large heterogeneity between studies and immediate medical attention is not always needed. However, current studies have several methodological shortcomings and standardization of image acquisition, assessment, and reporting is needed for future studies. When cone-beam computed tomographies are indicated for children and adolescents, they should be assessed for any incidental findings by either specialist oral and maxillofacial radiologists or dentists with appropriate training and experience.

## Supplementary Information

Below is the link to the electronic supplementary material.

Supplementary file 1 (DOCX 126 KB)

## Data Availability

All data have been made available through Zenodo (10.5281/zenodo.10854625).
