# Supplementary material for: Incidental findings from cone-beam computed tomography in children and adolescents: a systematic review
Source: Eur Arch Paediatr Dent. 2025 Jan 17;26(5):877–89. doi: 10.1007/s40368-025-00999-7 (PMC12532655; doi:10.1007/s40368-025-00999-7)
Supplement: Supplementary file 1 — (DOCX 126 KB) [file 40368_2025_999_MOESM1_ESM.docx]

**Incidental findings from cone-beam computed tomography in children and adolescents: a systematic review**

**SUPPLEMENTARY MATERIAL**

**Supplement.** Additional review methods and deviations from review protocol.

**Additional methods**

- Raw data available for two studies (provided for previous reviews) were analyzed by calculating summary statistics.

**Deviations from protocol**

- Some studies reported on patient populations consisting of both underage and overage patients and could not be included outright. In all instances, all corresponding authors were contacted and the raw data were attempted to be acquired to include only the subsample of underage patients. When this was not successful, the studies were excluded and all communication attempts were transparently reported.

**References**

Wan, X., Wang, W., Liu, J., & Tong, T. (2014). Estimating the sample mean and standard deviation from the sample size, median, range and/or interquartile range. BMC Medical Research Methodology, 14, 135.

**Supplementary Table 1.** Eligibility criteria for study inclusion according to the Participants – Exposure – Comparison – Outcome – Study design (PECOS) framework.

| **Component** | **Inclusion** | **Exclusion** |
| --- | --- | --- |
| Participants | Patients up to 18 years of age, of any sex or ethnicity with any kind of anamnestic or clinical justification deemed in need to receive a CBCT for diagnostic imaging reasons | Patients over 18 years of age |
| Exposure | CBCT for diagnostic imaging of the head / neck region | - Any kind of imaging modality except CBCT - Any kind of radiography used for therapeutic reasons (like cancer) - CBCT of any other area except the head / neck region |
| Comparison | Comparisons according to patient chracteristics |  |
| Outcome | Any incidental finding from CBCT images | Any other finding |
| Study design | Longitudinal or cross-sectional studies on humans | Animal studies, case series/reports, and non-clinical studies (i.e. cadaver studies) |

CBCT, cone-beam computed tomography.

**Supplementary Table 2**. Literature searches performed (last search date March 4^th^ 2024).

| **Database** | **Strategy** | **Hits** |
| --- | --- | --- |
| PubMed | (humans[mh] AND (minors[tiab] OR boy*[tiab] OR girl* OR kid*[tiab] OR child*[tiab] OR schoolchild*[tiab] OR school child[tiab] OR school child*[tiab] OR adolescen*[tiab] OR juvenil*[tiab] OR youth*[tiab] OR teen*[tiab] OR under*age*[tiab] OR pubescen*[tiab] OR pediatrics[mh] OR pediatric*[tiab] OR paediatric*[tiab] OR peadiatric*[tiab]) AND ("cone beam" OR CBCT OR (Cone-Beam Computed Tomography[MeSH])) AND (accidential* OR incidental* OR random* OR collateral* OR ancillar*) AND (finding* found* OR observ* OR detect* OR abnormal*)) | 41 |
| Embase | (toddler* OR boy OR boys OR boyhood OR girl* OR kid OR kids OR child OR child* OR children* OR schoolchild* OR schoolchild OR adolescen* OR juvenil* OR youth* OR teen* OR pubescen* OR pediatric* OR paediatric* OR peadiatric*) AND ("cone beam" OR CBCT) AND (accidential* OR incidental* OR random* OR collateral* OR ancillar*) AND (finding* found* OR observ* OR detect* OR abnormal*) | 197 |
| Scopus | Same as Embase | 60 |
| Web of Science | Same as Embase | 185 |
| CDSR | Same as Embase | 1 |
| CENTRAL | Same as Embase | 77 |
| DARE | Same as Embase | 0 |
| VHL | Same as Embase | 142 |
| **Sum before de-duplication** | | 550 |
| **Sum after de-duplication** | | 263 |

CDSR, Cochrane Database of Systematic Reviews; CENTRAL, Central Register of Controlled Trials; DARE, Database of Abstracts of Reviews of Effects; VHL, Virtual Health Library.

**Supplementary Table 3.** Comparison of the original Joanna Briggs institute and the risk of bias tool used in the present study.

| **Joanna Briggs Institute tool** | **Tool used in this study** |
| --- | --- |
| **Item** | **Item** |
| Was the sample frame appropriate to address the target population? | Are details given about the origin of the analyzed sample? |
|  | Is the acquisition timeframe of included CBCT images given? |
| Were study participants sampled in an appropriate way? | According to eligibility criteria, are included patients representative of the general healthy population, the target population of interest? |
|  | Were included cases selected consecutively or randomly? |
| Was the response rate adequate, and if not, was the low response rate managed appropriately? | - |
| Were the study subjects and the setting described in detail? | Is the age of included patients reported? |
|  | Is the sex of included patients reported? |
|  | Is the ethnicity of included patients reported? |
|  | Is the reason for acquisition of CBCT images reported? |
|  | Are inclusion / exclusion criteria of eligible patients reported? |
|  | According to the reason for CBCT acquisition, are included patients representative of the general population? |
|  | Do the authors state which area was included in the CBCT? |
|  | Are technical details about the acquired CBCT image given? (FOV, voxel, time, etc) |
| Were valid methods used for the identification of the condition? | Are CBCT images evaluated by an experienced certified radiologist? |
|  | Are CBCT assessors unaware of the reason for CBCT acquisition? |
| Was the condition measured in a standard, reliable way for all participants? | Have efforts been put into place to make sure optimal assessing conditions exist and errors are minimized? |
|  | Were all CBCT images assessed under the same conditions? |
|  | Have CBCT images been assessed from at least 2 assessors independently? |
|  | Have findings been reported on both patient level and CBCT image level, if applicable (multiple findings per image clarification)? |
| Was the data analysis conducted with sufficient coverage of the identified sample? | In case categorizations / poolings of findings have been made, have also separate individual findings been reported? |
|  | Have all possible findings that could be found in the particular CBCT image are been reported? |
| Was the sample size adequate? | Has an appropriately large sample been analyzed? |
| Was there appropriate statistical analysis? | Has the prevalence of observed findings been assessed? |

CBCT, cone beam computed tomography

**Supplementary Table 4**. Hits identified from the literature search together with their inclusion / exclusion status (with reasons).

| **Nr** | **Paper** | **Status** |
| --- | --- | --- |
| 1 | {Nct} Cognitively-Based Compassion Training for Breast Cancer Survivors. https://clinicaltrialsgov/show/NCT03305952. 2017. | Excluded by title |
| 2 | {Nct} Study to Compare the Efficacy of Cognitive-behavioral Couple Therapy and Lidocaine for Provoked Vestibulodynia. https://clinicaltrialsgov/show/NCT01935063. 2013. | Excluded by title |
| 3 | Aljahani J, Alaklabi A, Almalki W, Alfaleh H, Alzahrani Y. Splenic artery arising from hepatic artery proper in a patient with celiacomesenteric trunk: a rare anatomical variant. Surgical and Radiologic Anatomy. 2019;41(11):1391-4. | Excluded by title |
| 4 | Almeida GM, Carvalho VHM, Silva ÉBP, Cançado MAF, Barroso LS, Queiroz EL, et al. Periradicular repair after single- and two-visit root canal treatments using ultrasonic irrigant activation and calcium hydroxide dressing of teeth with apical periodontitis: study protocol for randomized controlled trials. Trials. 2023;24(1):23-. | Excluded by title |
| 5 | Attar B, Naeini M, Abdinian M. Investigation of the effect of fibrin glue as a sealant in the unilateral alveolar bone grafting successes. Dental Research Journal. 2023;20(1):60. | Excluded by title |
| 6 | Burkon P, Selingerova I, Slavik M, Holanek M, Vrzal M, Coufal O, et al. Toxicity of external beam accelerated partial-breast irradiation (APBI) in adjuvant therapy of early-stage breast cancer: prospective randomized study. Radiation Oncology. 2024;19(1). | Excluded by title |
| 7 | Carter A, Mohamed A. Dento-skeletal effects of different rapid maxillary expanders for growing patients-which is better? Evid Based Dent. 2023;24(3):104-5. | Excluded by title |
| 8 | Choi R, Lee M, Choi JW, Kim HC. Safety of Radioembolization via the Cystic Artery in Patients with Hepatocellular Carcinoma and Parasitized Arterial Supply. Journal of Vascular and Interventional Radiology. 2023;34(10):1802-8. | Excluded by title |
| 9 | Dutta S, Batra P, Raghavan S, Sharma K, Talwar A, Arora A, et al. Comparative assessment of facemask therapy with and without skeletal anchorage in growing Class III patients with unilateral cleft lip and palate (UCLP): A single-center, prospective randomized clinical trial. Special care in dentistry : official publication of the American Association of Hospital Dentists, the Academy of Dentistry for the Handicapped, and the American Society for Geriatric Dentistry. 2023. | Excluded by title |
| 10 | Elheeny AAH, Tony GE. Two-Dimensional Radiographs and Cone-beam Computed Tomography Assessment of Concentrated Growth Factor and Platelet-Rich Fibrin Scaffolds in Regenerative Endodontic Treatment of Immature Incisors with Periapical Radiolucency: A Randomized Clinical Trial. J Endod. 2024. | Excluded by title |
| 11 | Elsayed HS, El-Beialy AR, Alshazly R, Almohammad A, Elazab K, El-Badawy R, et al. Implant-supported canine retraction using different reactivation intervals of elastomeric chains: A CBCT-based split-mouth randomized controlled trial. Dental Press Journal of Orthodontics. 2023;28(5). | Excluded by title |
| 12 | Habib A, Athanasiou AE, Makrygiannakis MA, Kaklamanos EG. Do the various interceptive treatment modalities improve the position of palatally displaced permanent canines? A meta-analysis. European Journal of Orthodontics. 2023;45(4):370-81. | Excluded by title |
| 13 | Janiani P, Ramakrishnan M. Canal transportation and centering ability of neoendo rotary files in deciduous teeth: An in vitro study using cone beam computed tomography. Journal of Population Therapeutics and Clinical Pharmacology. 2023;30(10):e19-e23. | Excluded by title |
| 14 | Jung W, Lee KE, Suh BJ, Seok H, Lee DW. Deep learning for osteoarthritis classification in temporomandibular joint. Oral Diseases. 2023;29(3):1050-9. | Excluded by title |
| 15 | Lejnieks M, Akota I, Jākobsone G, Neimane L, Radzins O, Uribe SE. Effect of 3D printed replicas on the duration of third molar autotransplantation surgery: A controlled clinical trial. Dental traumatology : official publication of International Association for Dental Traumatology. 2023. | Excluded by title |
| 16 | Li SWS, Roberts E, Hedrich C. Treatment and monitoring of SAPHO syndrome: A systematic review. RMD Open. 2023;9(4). | Excluded by title |
| 17 | Materni A, Pasquale C, Signore A, Benedicenti S, Amaroli A. Comparison between the Flapless Surgical Approach and a Novel Single Incision Access in Terms of Recovery Time and Comfort after Extraction of Impacted Inferior Third Molars: A Randomised, Blinded, Split-Mouth Controlled Clinical Trial. Journal of Clinical Medicine. 2023;12(5). | Excluded by title |
| 18 | Mazzeo E, Bruni A, lotti C, Frezza G, Meduri B, Guidi G, et al. Hypofractionated IGRT for prostate cancer: first report on toxicity of a phase III randomized trial. Radiotherapy and Oncology. 2023;182:S150-S1. | Excluded by title |
| 19 | Mordente CM, Oliveira DD, Palomo JM, Cardoso PA, Assis MAL, Zenóbio EG, et al. The effect of micro-osteoperforations on the rate of maxillary incisors' retraction in orthodontic space closure: a randomized controlled clinical trial. Progress in Orthodontics. 2024;25(1). | Excluded by title |
| 20 | Patel N, Ahmad Z, Shah S, Sharma A, Ehsan A, Singh H, et al. Effect of root canal taper on the ability of endodontically treated teeth using the trunatomy and protaper next file systems to resist fracture. Journal of Pharmacy and Bioallied Sciences. 2023;15(6):S1123-S5. | Excluded by title |
| 21 | Pitman J, Christiaens V, Callens J, Glibert M, Seyssens L, Blanco J, et al. Immediate implant placement with flap or flapless surgery: A systematic review and meta-analysis. J Clin Periodontol. 2023;50(6):755-64. | Excluded by title |
| 22 | Rouientan A, Khodaparast MB, Safi Y. Evaluation of diagnostic accuracy of cone beam computed tomography and multi-detector computed tomography for detection of anatomical variations in rhinoplasty. Head and Face Medicine. 2024;20(1). | Excluded by title |
| 23 | Santos LM, Medeiros YL, Souza BG, Pereira KAM, Marlière DAA. Solitary intraosseous myofibroma: a rare case diagnosed from mandibular fracture. Gen Dent. 2023;71(4):31-5. | Excluded by title |
| 24 | Schlieve T. Case reporting in oral and maxillofacial pathology: Requisite among rare diseases. Oral and Maxillofacial Surgery Cases. 2023;9(2). | Excluded by title |
| 25 | Shao-Feng W, Xian-Ju XIE, Li Z, Qiao C, Fei-Fei ZUO, Ya-Jie W, et al. Research on multi-class orthodontic image recognition system based on deep learning network model. Chinese Journal of Stomatology. 2023(12):561-8. | Excluded by title |
| 26 | Shi F, Chen H, Li X, Gao R, Li R. Effect of contracted endodontic access cavity and root canal preparation by One Curve on danger zone of mesial root canal in mandibular first molars. Journal of Prevention and Treatment for Stomatological Diseases. 2023;31(7):480-7. | Excluded by title |
| 27 | Sun X, Li B, Abula D, Wang L, Wang B, Wang Q, et al. 0.1% Nano-silver mediates PD-1/PD-L1 pathway and alleviates chronic apical periodontitis in rats. Odontology. 2023;111(1):154-64. | Excluded by title |
| 28 | Teixeira R, Massaro C, Garib D. Comparison of nasal cavity changes between the expander with differential opening and the fan-type expander: a secondary data analysis from an RCT. Clin Oral Investig. 2023;27(10):5999-6006. | Excluded by title |
| 29 | Teixeira R, Massaro C, Garib D. Vertical and sagittal changes produced by an expander with differential opening and fan-type expander: A post-hoc analysis of a randomised controlled trial. Journal of orthodontics. 2023:14653125231208465. | Excluded by title |
| 30 | Wang SF, Xie XJ, Zhang L, Chang S, Zuo FF, Wang YJ, et al. [Research on multi-class orthodontic image recognition system based on deep learning network model]. Zhonghua Kou Qiang Yi Xue Za Zhi. 2023;58(6):561-8. | Excluded by title |
| 31 | Wrzosek M, Wilczek K, Tusiewicz J, Piskorz M, Rozylo-Kalinowska I. Pneumatization of the articular eminence in cone-beam computed tomography: prevalence and characteristics-literature review. Folia Morphologica. 2023;82(2):242-7. | Excluded by title |
| 32 | Algharbi M, Bazargani F, Dimberg L. Do different maxillary expansion appliances influence the outcomes of the treatment? European Journal of Orthodontics. 2018;40(1):97-106. | Excluded; not relevant |
| 33 | Bartal G, Vano E. Dose optimization is easy and improves image quality: Con. CardioVascular and Interventional Radiology. 2015;38(3):S65-S6. | Excluded; not relevant |
| 34 | Costa ACF, Maia TAC, de Barros Silva PG, Abreu LG, Gondim DV, Santos PCF. Effects of low-level laser therapy on the orthodontic mini-implants stability: a systematic review and meta-analysis. Progress in Orthodontics. 2021;22(1). | Excluded; not relevant |
| 35 | Cui Y, Huang M, Zheng J, Li J, Liu H, Liang C. Assessments of Coronary Artery Visibility and Radiation Dose in Infants with Congenital Heart Disease on Cardiac 128-slice CT and on Cardiac 64-slice CT. Pediatric Cardiology. 2016;37(1):135-43. | Excluded; not relevant |
| 36 | Cummings S, Chambers DW. Diagnostic paths for a mouth-breathing patient. Am J Orthod Dentofacial Orthop. 2020;158(4):564-71.e2. | Excluded; not relevant |
| 37 | De Dea M, L Loizou C, Allen GM, Wilson DJ, Athanasou N, Uchihara Y, et al. Talonavicular ligament: prevalence of injury in ankle sprains, histological analysis and hypothesis of its biomechanical function. Br J Radiol. 2017;90(1071):20160816-. | Excluded; not relevant |
| 38 | Delrue S, De Foer B, Casselman J, Offeriers FE. A persistent stapedial artery in a child with trisomy 8. B-ENT. 2013:29. | Excluded; not relevant |
| 39 | Fan XC, Singh D, Ma LS, Piehslinger E, Huang XF, Rausch-Fan X. Is there an association between temporomandibular disorders and articular eminence inclination? A systematic review. Diagnostics. 2021;11(1). | Excluded; not relevant |
| 40 | González-Hernández E, Campos D, Diego-Pedro R, Romero R, Baños R, Negi LT, et al. Changes in the Semantic Construction of Compassion after the Cognitively-Based Compassion Training (CBCT<sup>®</sup>) in Women Breast Cancer Survivors. Span J Psychol. 2021;24:e34-e. | Excluded; not relevant |
| 41 | Guerreiro F, Janssens G, Seravalli E, Raaymakers B. Abdominal diameter changes in children during volumetric modulated arc therapy (VMAT): Is re-planning needed? Medical Physics. 2016;43(6 PART2):3420. | Excluded; not relevant |
| 42 | Guo XL, Li G, Zheng JQ, Ma RH, Liu FC, Yuan FS, et al. Accuracy of detecting vertical root fractures in non-root filled teeth using cone beam computed tomography: effect of voxel size and fracture width. International endodontic journal. 2019;52(6):887-98. | Excluded; not relevant |
| 43 | Huang L, Zou R, He J, Ouyang K, Piao Z. Comparing osteogenic effects between concentrated growth factors and the acellular dermal matrix. Braz Oral Res. 2018;32:e29-e. | Excluded; not relevant |
| 44 | Huijskens SC, van Dijk I, Visser J, Balgobind BV, Te Lindert D, Rasch CRN, et al. Abdominal organ position variation in children during image-guided radiotherapy. Radiat Oncol. 2018;13(1):173. | Excluded; not relevant |
| 45 | Juan L, Peng L, Mengjun W, Yandong M. Impact of Hyperbaric Oxygen on the Healing of Bone Tissues Around Implants. Implant dentistry. 2018;27(6):653-9. | Excluded; not relevant |
| 46 | Katz J, Underhill T. Arrested pneumatization of the sphenoid sinus. Quintessence Int. 2013;44(6):437-41. | Excluded; not relevant |
| 47 | Kirschneck C, Fanghaenel J, Wahlmann U, Wolf M, Roldan JC, Proff P. Interactive effects of periodontitis and orthodontic tooth movement on dental root resorption, tooth movement velocity and alveolar bone loss in a rat model. Annals of Anatomy-Anatomischer Anzeiger. 2017;210:32-43. | Excluded; not relevant |
| 48 | Kirschneck C, Meier M, Bauer K, Proff P, Fanghaenel J. Meloxicam medication reduces orthodontically induced dental root resorption and tooth movement velocity: a combined in vivo and in vitro study of dental-periodontal cells and tissue. Cell and Tissue Research. 2017;368(1):61-78. | Excluded; not relevant |
| 49 | Koch R, Shim L, Al-Jewair T. Rapid Maxillary Expansion May Improve Condyle-Fossa Relationship and Intercondylar Symmetry. Journal of Evidence-Based Dental Practice. 2021;21(1). | Excluded; not relevant |
| 50 | Krug R, Connert T, Beinicke A, Soliman S, Schubert A, Kiefner P, et al. When and how do endodontic specialists use cone-beam computed tomography? Aust Endod J. 2019;45(3):365-72. | Excluded; not relevant |
| 51 | Krüsi M, Eliades T, Papageorgiou SN. Are there benefits from using bone-borne maxillary expansion instead of tooth-borne maxillary expansion? A systematic review with meta-analysis. Progress in Orthodontics. 2019;20(1). | Excluded; not relevant |
| 52 | Kry SF, Jones J, Childress NL. Implementation and evaluation of an end-to-end IGRT test. Journal of Applied Clinical Medical Physics. 2012;13(5):46-53. | Excluded; not relevant |
| 53 | Linguraru MG, Sandberg JK, Jones EC, Summers RM. Assessing Splenomegaly. Automated Volumetric Analysis of the Spleen. Academic Radiology. 2013;20(6):675-84. | Excluded; not relevant |
| 54 | Long J, Whalen J, Yang D, Strickland A, Kent K, Seyyedi S, et al. Classification of Motion Artifact Severity in High-Resolution Peripheral Quantitative Computed Tomography Using Deep Convolutional Neural Network. Journal of Bone and Mineral Research. 2020;35(SUPPL 1):208. | Excluded; not relevant |
| 55 | Masand P, Jadhav S, Agrawal H, Noel C, Mery C, Molossi S. Myocardial bridging in a cohort of pediatric patients with anomalous aortic origin of coronary artery (AAOCA): “Double whammy”! Pediatric Radiology. 2018;48(1):S128. | Excluded; not relevant |
| 56 | Mashyakhy M, Chourasia HR, Halboub E, Roges RA, Gambarini G. Nonsurgical Management and 2-year Follow-up by means of Cone Beam Computed Tomography of an Invasive Cervical Resorption in a Molar. The journal of contemporary dental practice. 2018;19(9):1152-6. | Excluded; not relevant |
| 57 | Masoud AI, Jackson GW, Carley DW. Sleep and airway assessment: A review for dentists. Cranio. 2017;35(4):206-22. | Excluded; not relevant |
| 58 | Mathur VP, Dhillon JK, Logani A, Kalra G. Evaluation of indirect pulp capping using three different materials: A randomized control trial using cone-beam computed tomography. Indian Journal of Dental Research. 2016;27(6):623-9. | Excluded; not relevant |
| 59 | Medina-Marino A, Bezuidenhout D, Hosek S, Barnabas RV, Atujuna M, Bezuidenhout C, et al. The Community PrEP Study: a randomized control trial leveraging community-based platforms to improve access and adherence to pre-exposure prophylaxis to prevent HIV among adolescent girls and young women in South Africa-study protocol. Trials. 2021;22(1):489. | Excluded; not relevant |
| 60 | Mohammadi D, Mehran M, Frankenberger R, BabeveyNejad N, Banakar M, Haghgoo R. Comparison of apical debris extrusion during root canal preparation in primary molars using different file systems: an in vitro study. Australian endodontic journal : the journal of the Australian Society of Endodontology Inc. 2021. | Excluded; not relevant |
| 61 | Moraes JdO. Avaliação da qualidade do preparo de canais de dentes decíduos artificiais utilizando o sistema de rotação recíproca. 2015. p. 66-. | Excluded; not relevant |
| 62 | Moshi FV, Kibusi SM, Fabian FM. The impact of community based continuous training project on improving couples' knowledge on birth preparedness and complication readiness in rural setting Tanzania; A controlled quasi-experimental study. PLoS One. 2021;16(1):e0244845-e. | Excluded; not relevant |
| 63 | Mossaz J, Suter VGA, Katsaros C, Bornstein MM. [Supernumerary teeth in the maxilla and mandible-an interdisciplinary challenge. Part 1: epidemiology, etiology, classification and associated complications]. Swiss Dent J. 2016;126(2):131-49. | Excluded; not relevant |
| 64 | Mounir M, Atef M, Abou-Elfetouh A, Hakam MM. Titanium and polyether ether ketone (PEEK) patient-specific sub-periosteal implants: two novel approaches for rehabilitation of the severely atrophic anterior maxillary ridge. International Journal of Oral and Maxillofacial Surgery. 2018;47(5):658-64. | Excluded; not relevant |
| 65 | Mudimu E, Sardinia J, Momin S, Medina-Marino A, Bezuidenhout C, Bekker LG, et al. Incremental costs of integrated PrEP provision and effective use counselling in community-based platforms for adolescent girls and young women in South Africa: an observational study. J Int AIDS Soc. 2022;25(2):e25875. | Excluded; not relevant |
| 66 | Naoumova J. Interceptive Treatment Of Palatally Displaced Canines. Swed Dent J Suppl. 2014(234):7-118. | Excluded; not relevant |
| 67 | Noronha Oliveira M, Rau LH, Marodin A, Corrêa M, Corrêa LR, Aragones A, et al. Ridge Preservation After Maxillary Third Molar Extraction Using 30% Porosity PLGA/HA/β-TCP Scaffolds With and Without Simvastatin: A Pilot Randomized Controlled Clinical Trial. Implant dentistry. 2017;26(6):832-40. | Excluded; not relevant |
| 68 | Orozco EIF, Toia CC, Cavalli D, Khoury RD, Cardoso F, Bresciani E, et al. Effect of passive ultrasonic activation on microorganisms in primary root canal infection: a randomized clinical trial. Journal of applied oral science : revista FOB. 2020;28:e20190100. | Excluded; not relevant |
| 69 | Pace T, Negi L, Donaldson-Lavelle B, Ozawa-de Silva B, Reddy S, Cole S, et al. Cognitively-Based Compassion Training reduces peripheral inflammation in adolescents in foster care with high rates of early life adversity. BMC complementary and alternative medicine. 2012;12. | Excluded; not relevant |
| 70 | Pace T, Negi LT, Dodson-Lavelle B, Ozawa-De Silva B, Reddy SD, Cole SP, et al. Engagement with cognitively-based compassion training is associated with reduced salivary C-reactive protein and cortisol from before to after training in foster care program adolescents. Brain, behavior, and immunity. 2012;26:S43. | Excluded; not relevant |
| 71 | Pace TW, Negi LT, Dodson-Lavelle B, Ozawa-de Silva B, Reddy SD, Cole SP, et al. Engagement with Cognitively-Based Compassion Training is associated with reduced salivary C-reactive protein from before to after training in foster care program adolescents. Psychoneuroendocrinology. 2013;38(2):294-9. | Excluded; not relevant |
| 72 | Poehlmann-Tynan J, Engbretson A, Vigna AB, Weymouth LA, Burnson C, Zahn-Waxler C, et al. Cognitively-Based Compassion Training for parents reduces cortisol in infants and young children. Infant Ment Health J. 2020;41(1):126-44. | Excluded; not relevant |
| 73 | Qian WL, Jiang Y, Liu X, Guo YK, Li Y, Tang X, et al. Distinguishing cardiac myxomas from cardiac thrombi by a radiomics signature based on cardiovascular contrast-enhanced computed tomography images. BMC Cardiovascular Disorders. 2021;21(1). | Excluded; not relevant |
| 74 | Radhika E, Reddy ER, Rani ST, Kumar LV, Manjula M, Mohan TA. Cone Beam Computed Tomography Evaluation of Hand Nickel-Titanium K-Files and Rotary System in Primary Teeth. Pediatric Dentistry. 2017;39(4):319-23. | Excluded; not relevant |
| 75 | Sarabia-Estrada R, Ruiz-Valls A, Shah SR, Ahmed AK, Ordonez AA, Rodriguez FJ, et al. Effects of primary and recurrent sacral chordoma on the motor and nociceptive function of hindlimbs in rats: An orthotopic spine model. Journal of Neurosurgery: Spine. 2017;27(2):215-26. | Excluded; not relevant |
| 76 | Shamu S, Chasela C, Slabbert J, Farirai T, Guloba G, Nkhwashu N. Social franchising of community-based HIV counselling and testing services to increase HIV testing and linkage to care in Tshwane, South Africa: study protocol for a non-randomised implementation trial. BMC Public Health. 2020;20(1):118-. | Excluded; not relevant |
| 77 | Shamu S, Farirai T, Kuwanda L, Slabbert J, Guloba G, Khupakonke S, et al. Comparison of community-based HIV counselling and testing (CBCT) through index client tracing and other modalities: Outcomes in 13 South African high HIV prevalence districts by gender and age. PLoS One. 2019;14(9):e0221215. | Excluded; not relevant |
| 78 | Sharif MO, Horner K, Chadwick S, West C. Susuk charms? A case report. Br Dent J. 2013;215(1):13-5. | Excluded; not relevant |
| 79 | Shnaider P, Sijercic I, Wanklyn SG, Suvak MK, Monson CM. The Role of Social Support in Cognitive-Behavioral Conjoint Therapy for Posttraumatic Stress Disorder. Behav Ther. 2017;48(3):285-94. | Excluded; not relevant |
| 80 | Siegel M, Schmidt B, Giraldo JCR. Single energy CT with tin filtration for pulmonary nodule detection: Phantom study using exposures as low as 10 microgray. Pediatric Radiology. 2017;47:S155-S6. | Excluded; not relevant |
| 81 | Silva SR, Silva JD, Schnaider TB, Veiga DF, Novo NF, Mesquita M, et al. The use of a biocompatible cement in endodontic surgery. A randomized clinical trial 1. Acta cirurgica brasileira. 2016;31(6):422‐7. | Excluded; not relevant |
| 82 | Sun Q, Lee JS, Kim O, Kim Y. Primordial odontogenic tumor: a case report and literature review. Diagn Pathol. 2019;14(1):92. | Excluded; not relevant |
| 83 | Suomi A, Evans L, Rodgers B, Taplin S, Cowlishaw S. Couple and family therapies for post-traumatic stress disorder (PTSD). Cochrane Database of Systematic Reviews. 2019;2019(12). | Excluded; not relevant |
| 84 | Titanji BK, Tejani M, Farber EW, Mehta CC, Pace TW, Meagley K, et al. Cognitively Based Compassion Training for HIV Immune Nonresponders - An Attention-Placebo Randomized Controlled Trial. Journal of Acquired Immune Deficiency Syndromes. 2022;89(3):340-8. | Excluded; not relevant |
| 85 | Tøndel H, Lund J, Lydersen S, Wanderås AD, Aksnessæther BY, Jensen CA, et al. Dose to penile bulb is not associated with erectile dysfunction 18months post radiotherapy: A secondary analysis of a randomized trial. Clinical and Translational Radiation Oncology. 2018;13:50-6. | Excluded; not relevant |
| 86 | Walsh T, Macey R, Riley P, Glenny A-M, Schwendicke F, Worthington HV, et al. Imaging modalities to inform the detection and diagnosis of early caries. Cochrane Database of Systematic Reviews. 2021(3). | Excluded; not relevant |
| 87 | Wang R, Li F, Chen S, Liu D, Yang R. A New Method for Anterior Boundary Demarcation of the Nasopharynx in Three-Dimensional Analysis. Journal of Craniofacial Surgery. 2022;33(2):400-3. | Excluded; not relevant |
| 88 | Wang X, MacDougall RD, Chen P, Bouman CA, Warfield SK. Physics-based iterative reconstruction for dual-source and flying focal spot computed tomography. Medical Physics. 2021;48(7):3595-613. | Excluded; not relevant |
| 89 | Wolf TG, Fischer F, Schulze RKW. Correlation of objective image quality and working length measurements in different CBCT machines: An ex vivo study. Scientific reports. 2020;10(1):19414. | Excluded; not relevant |
| 90 | Xie L, Tang W, Izadikhah I, Zhao Z, Zhao Y, Li H, et al. Development of a multi-stage model for intelligent and quantitative appraising of skeletal maturity using cervical vertebras cone-beam CT images of Chinese girls. Int J Comput Assist Radiol Surg. 2022;17(4):761-73. | Excluded; not relevant |
| 91 | Xu Y, Sun J, Xu Y, Yu Y. Introduction of Accurate Measurement of the Alveolar Bone Height in Bone Grafting Area after Sinus Lift. Current medical imaging. 2022. | Excluded; not relevant |
| 92 | Yao W, Farr JB. A multiscale filter for noise reduction of low-dose cone beam projections. Physics in medicine and biology. 2015;60(16):6515-30. | Excluded; not relevant |
| 93 | Yatabe M, Gomes L, Ruellas AC, Lopinto J, Macron L, Paniagua B, et al. Challenges in measuring angles between craniofacial structures. Journal of applied oral science : revista FOB. 2019;27:e20180380. | Excluded; not relevant |
| 94 | Zaghloul MS, Mousa AG, Eldebawy E, Attalla E, Shafik H, Ezzat S. Comparison of electronic portal imaging and cone beam computed tomography for position verification in children. Clin Oncol (R Coll Radiol). 2010;22(10):850-61. | Excluded; not relevant |
| 95 | {Nct} Assessment of Extraction of Primary Canines in Treating Mesioangular Displaced Permanent Canines. https://clinicaltrialsgov/show/NCT03684525. 2018. | Excluded; no fulltext available |
| 96 | Batista KBDSL, Lima T, Palomares N, Carvalho FdA, Quintão C, Miguel JAM, et al. Herbst appliance with skeletal anchorage versus dental anchorage in adolescents with Class II malocclusion: study protocol for a randomised controlled trial. Trials. 2017;18(1):564-. | Excluded; no fulltext available |
| 97 | Caldeira AV, Amorim Souza AC, Trindade Miguel MA, Ribeiro IP, de Almeida Junior LA, Sakai VT, et al. Surgical and orthodontic treatment after incidental radiographic discovery of a severely infraoccluded primary molar. General Dentistry. 2019;67(6):72-5. | Excluded; no fulltext available |
| 98 | Feng J-k, Gao J-h. Fenestration and dehiscence in anterior alveolar bone of 52 adolescent patients with skeletal class II and high-angle malocclusion assessed with cone-beam computed tomography. Jiepou Xuebao. 2019;50(2):241-4. | Excluded; no fulltext available |
| 99 | Ramesh A, Pabla T. Incidental finding on dental radiographs: benign fibro-osseous lesions of the jaws. J Mass Dent Soc. 2008;57(3):60-1. | Excluded; no fulltext available |
| 100 | Ramesh A, Pabla T. Incidental findings on dental radiographs: dentigerous cyst. J Mass Dent Soc. 2009;58(2):42-. | Excluded; no fulltext available |
| 101 | Tucunduva RMA. Estudo do forame de Huschke em exames de tomografia computadorizada de feixe cônico. 2015. p. 77-. | Excluded; no fulltext available |
| 102 | Deng Y, Sun Y, Xu T. Evaluation of root resorption after comprehensive orthodontic treatment using cone beam computed tomography (CBCT): a meta-analysis. BMC Oral Health. 2018;18(1):116. | Excluded; review |
| 103 | Di Carlo G, Saccucci M, Ierardo G, Luzzi V, Occasi F, Zicari AM, et al. Rapid Maxillary Expansion and Upper Airway Morphology: A Systematic Review on the Role of Cone Beam Computed Tomography. Biomed Research International. 2017;2017. | Excluded; review |
| 104 | Edwards R, Altalibi M, Flores-Mir C. The frequency and nature of incidental findings in cone-beam computed tomographic scans of the head and neck region A systematic review. Journal of the American Dental Association. 2013;144(2):161-70. | Excluded; review |
| 105 | {Nct} Validation of X-ray Protocols in Cleft Children. https://clinicaltrialsgov/show/NCT05351372. 2022. | Excluded; not healthy patients |
| 106 | Altun BD, Dumlu A. Evaluation of maxillary sinus volumes and pathologies in children with and without cleft lip and palate using cone beam computed tomography. Orthodontics & Craniofacial Research. 2023. | Excluded; not healthy patients |
| 107 | Attar BM, Naghdi N, Sh ME, Mehdizadeh M. Chin Symphysis Bone, Allograft, and Platelet-Rich Fibrin: Is the Combination Effective in Repair of Alveolar Cleft? Journal of Oral and Maxillofacial Surgery. 2017;75(5):1026-35. | Excluded; not healthy patients |
| 108 | Bahouth S, Zhang W, Masand P, Jadhav S, Lyons K, Krishnamurthy R. Use of CT and MR in neonatal and infantile congenital heart disease: Change in practice patterns with advent of new generation CT scanner. Pediatric Radiology. 2015;45:S81-S2. | Excluded; not healthy patients |
| 109 | Baues C, Görgen H, Semrau R, Nast-Kolb B, Assenmacher K, Celik E, et al. Volumetric assessment of mediastinal lymphoma masses in Hodgkin lymphoma. Leuk Lymphoma. 2019;60(13):3244-50. | Excluded; not healthy patients |
| 110 | Bayrakdar I, Yasa Y, Duman Ş, Karaturgut UE, Ocak A, Günen Yılmaz S. Cone beam computed tomography evaluation of ponticulus posticus in patients with cleft lip and palate: a retrospective radio-anatomic study. Folia morphologica. 2018;77(1):72‐8. | Excluded; not healthy patients |
| 111 | Celikoglu M, Buyuk SK, Sekerci AE, Ersoz M, Celik S, Sisman Y. Facial soft-tissue thickness in patients affected by bilateral cleft lip and palate: a retrospective cone-beam computed tomography study. Am J Orthod Dentofacial Orthop. 2014;146(5):573-8. | Excluded; not healthy patients |
| 112 | Contreras V, Carrasco-Labra A, Andrews N, Brignardello-Petersen R, Pantoja R. [Cross-sectional study of deviated nasal septum in unilaterally operated fissures]. Cirugia pediatrica : organo oficial de la Sociedad Espanola de Cirugia Pediatrica. 2012;25(2):75-7. | Excluded; not healthy patients |
| 113 | de Almeida AM, Ozawa TO, Alves ACM, Janson G, Lauris JRP, Ioshida MSY, et al. Slow versus rapid maxillary expansion in bilateral cleft lip and palate: a CBCT randomized clinical trial. Clin Oral Investig. 2017;21(5):1789-99. | Excluded; not healthy patients |
| 114 | de Ruiter A, Janssen N, van Es R, Frank M, Meijer G, Koole R, et al. Micro-structured Beta-Tricalcium Phosphate for Repair of the Alveolar Cleft in Cleft Lip and Palate Patients: A Pilot Study. Cleft Palate Craniofac J. 2015;52(3):336-40. | Excluded; not healthy patients |
| 115 | Deçolli Y, Nemţoi A, Susanu S, Haba D, Petcu A. A software tool used in 3D evaluation of the alveolar bone defect in bilateral cleft lip and palate patients. Rev Med Chir Soc Med Nat Iasi. 2014;118(3):841-6. | Excluded; not healthy patients |
| 116 | Figueiredo DSF, Cardinal L, Bartolomeo FUC, Palomo JM, Horta MCR, Andrade I, Jr., et al. Effects of rapid maxillary expansion in cleft patients resulting from the use of two different expanders. Dental press journal of orthodontics. 2016;21(6):82-90. | Excluded; not healthy patients |
| 117 | Garib D, Lauris RC, Calil LR, Alves AC, Janson G, De Almeida AM, et al. Dentoskeletal outcomes of a rapid maxillary expander with differential opening in patients with bilateral cleft lip and palate: A prospective clinical trial. Am J Orthod Dentofacial Orthop. 2016;150(4):564-74. | Excluded; not healthy patients |
| 118 | Ghoneima A, Allam E, Kula K. Effects of Primary Alveolar Grafting on Alveolar Bone Thickness in Patients With Cleft Lip and Palate. J Craniofac Surg. 2017;28(5):1337-41. | Excluded; not healthy patients |
| 119 | Kuijpers MAR, Pazera A, Admiraal RJ, Bergé SJ, Vissink A, Pazera P. Incidental findings on cone beam computed tomography scans in cleft lip and palate patients. Clinical Oral Investigations. 2014;18(4):1237-44. | Excluded; not healthy patients |
| 120 | Kula K, Hale LN, Ghoneima A, Tholpady S, Starbuck JM. Cone-Beam Computed Tomography Analysis of Mucosal Thickening in Unilateral Cleft Lip and Palate Maxillary Sinuses. Cleft Palate Craniofac J. 2016;53(6):640-8. | Excluded; not healthy patients |
| 121 | Montoya JC, Eckel LJ, DeLone DR, Kotsenas AL, Diehn FE, Yu L, et al. Low-dose CT for craniosynostosis: Preserving diagnostic benefit with substantial radiation dose reduction. American Journal of Neuroradiology. 2017;38(4):672-7. | Excluded; not healthy patients |
| 122 | Santos G, Ickow I, Job J, Brooker JE, Dvoracek LA, Rigby E, et al. Cone-Beam Computed Tomography Incidental Findings in Individuals With Cleft Lip and Palate. Cleft Palate-Craniofacial Journal. 2020;57(4):404-11. | Excluded; not healthy patients |
| 123 | Saruhan N, Ertas U. Evaluating of Platelet-Rich Fibrin in the Treatment of Alveolar Cleft With Iliac Bone Graft By Means of Volumetric Analysis. Journal of Craniofacial Surgery. 2018;29(2):322-6. | Excluded; not healthy patients |
| 124 | Sobti G, Chaudhry A, Thanvi J, Gaurav I, Shekhawat C, Banerjee D, et al. Co-Occurrence of Taurodontism in Nonsyndromic Cleft Lip and Palate Patients in Subset of Indian Population: A Case-Control Study Using CBCT. Cleft Palate-Craniofacial Journal. 2022. | Excluded; not healthy patients |
| 125 | Al Qabbani A, Al Kawas S, Razak NHA, Al Bayatti SW, Enezei HH, Samsudin AR, et al. Three-Dimensional Radiological Assessment of Alveolar Bone Volume Preservation Using Bovine Bone Xenograft. Journal of Craniofacial Surgery. 2018;29(2):E203-E9. | Excluded; not on children |
| 126 | Alagl A, Bedi S, Hassan K, AlHumaid J. Use of platelet-rich plasma for regeneration in non-vital immature permanent teeth: Clinical and cone-beam computed tomography evaluation. Journal of International Medical Research. 2017;45(2):583-93. | Excluded; not on children |
| 127 | Alcântara CEP, Castro MAA, Noronha MSd, Martins-Junior PA, Mendes RdM, Caliari MV, et al. Hyaluronic acid accelerates bone repair in human dental sockets: a randomized triple-blind clinical trial. Braz Oral Res. 2018;32:e84-e. | Excluded; not on children |
| 128 | Aminabadi NA, Behroozian A, Talatahari E, Samiei M, Sadigh-Eteghad S, Shirazi S. Does prenatal restraint stress change the craniofacial growth pattern of rat offspring? European journal of oral sciences. 2016;124(1):17-25. | Excluded; not on humans |
| 129 | Anitua E, Murias-Freijo A, Hamdan Alkhraisat M, Orive G. Clinical, radiographical, and histological outcomes of plasma rich in growth factors in extraction socket: a randomized controlled clinical trial. Clinical Oral Investigations. 2015;19(3):589-600. | Excluded; not on children |
| 130 | Aydin H, Mobaraki S. Comparison of root and canal anatomy of taurodont and normal molar teeth: A retrospective cone-beam computed tomography study. Archives of Oral Biology. 2021;130. | Excluded; not on children |
| 131 | Bagourd T, Varazzani A, Dugast S, Guyonvarc'h P, Corre P, Bertin H. Radiological evaluation of inferior alveolar nerve displacement after removal of impacted mandibular third molars prior to sagittal split osteotomy. Journal of stomatology, oral and maxillofacial surgery. 2023;124(6S2):101658. | Excluded; not on children |
| 132 | Bindakhil M, Shanti RM, Mupparapu M. Raloxifene-induced osteonecrosis of the jaw (MRONJ) with no exposure to bisphosphonates: clinical and radiographic findings. Quintessence Int. 2021;0(0):2-7. | Excluded; not on children |
| 133 | Chawla R, Garcha P, Lyne A, Sheriteh Z. Evidence that ectopic upper third molars can result in upper second molar impactions: A case series. J Orthod. 2023:14653125231178039-. | Excluded; not on children |
| 134 | Cömert Kiliç S, Güngörmüş M, Sümbüllü MA. Is Arthrocentesis Plus Platelet-Rich Plasma Superior to Arthrocentesis Alone in the Treatment of Temporomandibular Joint Osteoarthritis? A Randomized Clinical Trial. Journal of Oral and Maxillofacial Surgery. 2015;73(8):1473-83. | Excluded; not on children |
| 135 | Cömert Kılıç S. Does glucosamine, chondroitin sulfate, and methylsulfonylmethane supplementation improve the outcome of temporomandibular joint osteoarthritis management with arthrocentesis plus intraarticular hyaluronic acid injection. A randomized clinical trial. Journal of Cranio-Maxillofacial Surgery. 2021;49(8):711-8. | Excluded; not on children |
| 136 | Enciso R, Shigeta Y, Nguyen M, Clark GT. Comparison of cone-beam computed tomography incidental findings between patients with moderate/severe obstructive sleep apnea and mild obstructive sleep apnea/healthy patients. Oral Surgery, Oral Medicine, Oral Pathology and Oral Radiology. 2012;114(3):373-81. | Excluded; not on children |
| 137 | Gilbert SR, Estep P, Smoot M, Smith K, Brooks R, Jaudon H, et al. Obesity alters growth plate thickness and strength in immature rats. Journal of Orthopaedic Research. 2016;34. | Excluded; not on humans |
| 138 | Gizani S, Seremidi K, Gkourtsogianni S, Mitsea A. Awareness and practice of dentomaxillofacial imaging among paediatric dentists: a questionnaire survey of members of the European Academy of Paediatric Dentistry. Oral Radiol. 2023;39(3):576-87. | Excluded; not on children |
| 139 | Handem RH. Prescrição tomográfica volumétrica e a responsabilidade do radiologista na interpretação das imagens. 2016. p. 86-. | Excluded; not on children |
| 140 | Kato CN, Tavares NP, Barra SG, Amaral TM, Brasileiro CB, Abreu LG, et al. Digital panoramic radiography and cone-beam CT as ancillary tools to detect low bone mineral density in post-menopausal women. Dento maxillo facial radiology. 2019;48(2):20180254. | Excluded; not on children |
| 141 | Langella J, Finkelman MD, Alon E, Fida Z, Martin A, Amato R. Incidental Findings in Small Field of View Cone-beam Computed Tomography Scans, Part 2: Interpretation with Aid of a Checklist. Journal of endodontics. 2023;49(4):390-4. | Excluded; not on children |
| 142 | Linderup BW, Küseler A, Jensen J, Cattaneo PM. A novel semiautomatic technique for volumetric assessment of the alveolar bone defect using cone beam computed tomography. Cleft Palate Craniofac J. 2015;52(3):e47-55. | Excluded; not on children |
| 143 | Pette GA, Norkin FJ, Ganeles J, Hardigan P, Lask E, Zfaz S, et al. Incidental findings from a retrospective study of 318 cone beam computed tomography consultation reports. International Journal of Oral and Maxillofacial Implants. 2012;27(3):595-603. | Excluded; not on children |
| 144 | Qabbani AA, Razak NHA, Kawas SA, Sheikh Abdul Hamid S, Wahbi S, Samsudin AR. The Efficacy of Immediate Implant Placement in Extraction Sockets for Alveolar Bone Preservation: a Clinical Evaluation Using Three-Dimensional Cone Beam Computerized Tomography and Resonance Frequency Analysis Value. Journal of craniofacial surgery. 2017;28(4):e318‐e25. | Excluded; not on children |
| 145 | Silveira BT, Fernandes KS, Trivino T, Dos Santos LYF, de Freitas CF. Assessment of the relationship between size, shape and volume of the sella turcica in class II and III patients prior to orthognathic surgery. Surg Radiol Anat. 2020;42(5):577-82. | Excluded; not on children |
| 146 | Štoković N, Trkulja V, Dumić-Čule I, Čuković-Bagić I, Lauc T, Vukičević S, et al. Sphenoid sinus types, dimensions and relationship with surrounding structures. Annals of Anatomy. 2016;203:69-76. | Excluded; not on children |
| 147 | Streckbein P, Jäckel S, Malik CY, Obert M, Kähling C, Wilbrand JF, et al. Reconstruction of critical-size mandibular defects in immunoincompetent rats with human adipose-derived stromal cells. Journal of Cranio-Maxillofacial Surgery. 2013;41(6):496-503. | Excluded; not on humans |
| 148 | Van Gorp G, Maes A, Lambrechts M, Jacobs R, Declerck D. Is use of CBCT without proper training justified in paediatric dental traumatology? An exploratory study. BMC Oral Health. 2023;23(1):270. | Excluded; not on children |
| 149 | Xu L, Li Y, Mei L, Qi H, Fang J, Li Y. Local injection of abaloparatide promotes mandibular condyle lengthening in adolescent rats via enhancing chondrogenesis and ossification. J Oral Rehabil. 2024;51(2):380-93. | Excluded; not on humans |
| 150 | Yang P, Qu X, Qi S, Li G, Wang S. Oral administration of inorganic nitrate alleviated biological damage induced by cone-beam computed tomography examination in Wistar rats. Nitric Oxide - Biology and Chemistry. 2022;122-123:19-25. | Excluded; not on humans |
| 151 | Zou Y, Lin H, Cai J, Xie Q, Chen W, Lu Y-G, et al. Effects of functional mandibular lateral shift on craniofacial growth and development in growing rats. Journal of Oral Rehabilitation. 2022. | Excluded; not on humans |
| 152 | Altindag A, Avsever H, Borahan O, Akyol M, Orhan K. Incidental Findings in Cone-Beam Computed Tomographic Images: Calcifications in Head and Neck Region. Balk J Dent Med, 2017; 100-107. | Excluded; missing data; contact attempted |
| 153 | Bayrak S, Bulut DG, Çakmak ESK, Orhan K. Cone beam computed tomographic evaluation of intracranial physiologic calcifications. Journal of Craniofacial Surgery. 2019;30(2):510-3. | Excluded; mixed sample; contact attempted |
| 154 | Borghesi A, Michelini S, Zigliani A, Tonni I, Maroldi R. Three-rooted maxillary first premolars incidentally detected on cone beam CT: an in vivo study. Surgical and Radiologic Anatomy. 2019;41(4):461-8. | Excluded; mixed sample; contact attempted |
| 155 | Braun MJ, Rauneker T, Dreyhaupt J, Hoffmann TK, Luthardt RG, Schmitz B, et al. Dental and Maxillofacial Cone Beam CT—High Number of Incidental Findings and Their Impact on Follow-Up and Therapy Management. Diagnostics. 2022;12(5). | Excluded; mixed sample; contact attempted |
| 156 | Bruno G, Stefani AD, Benetazzo C, Cavallin F, Gracco A. Changes in nasal septum morphology after rapid maxillary expansion: a Cone-Beam Computed Tomography study in pre-pubertal patient. Dental Press J Orthod. 2020;25(5):51-6. | Excluded; missing data; contact attempted |
| 157 | Çaǧlayan F, Tozoǧlu U. Incidental findings in the maxillofacial region detected by cone beam CT. Diagnostic and Interventional Radiology. 2012;18(2):159-63. | Excluded; mixed sample; contact attempted |
| 158 | Cha J-Y, Mah J, Sinclair P. Incidental findings in the maxillofacial area with 3-dimensional cone-beam imaging. American Journal of Orthodontics and Dentofacial Orthopedics. 2007;132(1):7-14. | Excluded; mixed sample; contact attempted |
| 159 | Choi J-Y, Oh SH, Kim S-H, Ahn H-W, Kang Y-G, Choi Y-S, et al. Effectiveness of 2D radiographs in detecting CBCT-based incidental findings in orthodontic patients. Sci Rep. 2021;11(1):9280-. | Excluded; mixed sample; contact attempted |
| 160 | Drage N, Rogers S, Greenall C, Playle R. Incidental findings on cone beam computed tomography in orthodontic patients. Journal of Orthodontics. 2013;40(1):29-37. | Excluded; mixed sample; contact attempted |
| 161 | Edwards R, Alsufyani N, Heo G, Flores-Mir C. The frequency and nature of incidental findings in large-field cone beam computed tomography scans of an orthodontic sample. Progress in orthodontics. 2014;15(1):37. | Excluded; mixed sample; contact attempted |
| 162 | Evirgen S, Yuksel HT, Yuksel G, Kaki B, Kamburoglu K. Assessment of intravertebral pneumatocysts, degenerative joint disease, and ponticulus posticus in the cervical spine through cone beam computed tomography examination. Oral Surgery Oral Medicine Oral Pathology Oral Radiology. 2020;129(5):531-8. | Excluded; mixed sample; contact attempted |
| 163 | Gümrü B, Guldali M, Tarcin B, Idman E, Sertac Peker M. Evaluation of cone beam computed tomography referral profile: Retrospective study in a Turkish paediatric subpopulation. Eur J Paediatr Dent. 2021;22(1):66-70. | Excluded; missing data; contact attempted |
| 164 | Oliveira RdS, Peretto JT, Panzarella FK, Raitz R. Evaluation of Incidental Findings on Cone Beam Computed Tomography. Pesqui bras odontopediatria clín integr. 2019;19(1):4340-. | Excluded; mixed sample; contact attempted |
| 165 | Pazera P, Bornstein MM, Pazera A, Sendi P, Katsaros C. Incidental maxillary sinus findings in orthodontic patients: A radiographic analysis using cone-beam computed tomography (CBCT). Orthodontics and Craniofacial Research. 2011;14(1):17-24. | Excluded; mixed sample; contact attempted |
| 166 | Pliska B, DeRocher M, Larson BE. Incidence of significant findings on CBCT scans of an orthodontic patient population. Northwest Dent. 2011;90(2):12-6. | Excluded; mixed sample; contact attempted |
| 167 | Price JB, Thaw KL, Tyndall DA, Ludlow JB, Padilla RJ. Incidental findings from cone beam computed tomography of the maxillofacial region: a descriptive retrospective study. Clin Oral Implants Res. 2012;23(11):1261-8. | Excluded; mixed sample; contact attempted |
| 168 | Ritter L, Lutz J, Neugebauer J, Scheer M, Dreiseidler T, Zinser MJ, et al. Prevalence of pathologic findings in the maxillary sinus in cone-beam computerized tomography. Oral Surgery Oral Medicine Oral Pathology Oral Radiology and Endodontology. 2011;111(5):634-40. | Excluded; mixed sample; contact attempted |
| 169 | Shokri A, Mortazavi H, Baharvand M, Falah-Kooshki S, Ostovarrad F, Karimi A. Prevalence of incidental findings in paranasal sinuses using CBCT. Dental and Medical Problems. 2014;51(4):431-8. | Excluded; mixed sample; contact attempted |
| 170 | Thaw KL. Incidental findings from cone beam computed tomography of the maxillofacial region: a descriptive retrospective study. MSc Thesis, 2010, Chapel Hill. | Excluded; mixed sample; contact attempted |
| 171 | Theodoridis C, Vaitsidis Z, Angelopoulos C. Incidental findings on CBCT and classification according to their significance. Oral Surg Oral Med Oral Pathol Oral Radiol 2019;127(1):47. | Excluded; missing fulltext; contact attempted |
| 172 | Alam MK. Laser assisted orthodontic tooth movement in saudi population: A randomized clinical trial. Bangladesh Journal of Medical Science. 2019;18(2):385-90. | Excluded; no CBCT |
| 173 | Beyoğlu ÇA, Kendigelen P, Beyoğlu İ, Altındaş F, Kuruoğlu S, Kaya G. Does magnetic resonance imaging increase core body temperature in children? Results of the administration of propofol and ketofol: A randomized clinical study. Turkish Journal of Pediatrics. 2020;62(2):224-32. | Excluded; no CBCT |
| 174 | Fakhran S, Alhilali L, Sreedher G, Dohatcu AC, Lee S, Ferguson B, et al. Comparison of simulated cone beam computed tomography to conventional helical computed tomography for imaging of rhinosinusitis. Laryngoscope. 2014;124(9):2002-6. | Excluded; no CBCT |
| 175 | Hlongwa P, Moshaoa MAL, Musemwa C, Khammissa RAG. Incidental Pathologic Findings from Orthodontic Pretreatment Panoramic Radiographs. International Journal of Environmental Research and Public Health. 2023;20(4 C7 - 3479). | Excluded; no CBCT |
| 176 | Imanimoghaddam M, Tohidi E, Yazdi AA, Nikbakhsh E, Goudarzi F. Incidental findings in digital panoramic radiography of patients referred to mashhad dental school. Journal of Kerman University of Medical Sciences. 2021;28(1):43-55. | Excluded; no CBCT |
| 177 | Johnsen GF, Sundnes J, Wengenroth J, Haugen HJ. Methodology for Morphometric Analysis of Modern Human Contralateral Premolars. J Comput Assist Tomogr. 2016;40(4):617-25. | Excluded; no CBCT |
| 178 | Joshi M, Wu LP, Maharjan S, Regmi MR. Sagittal lip positions in different skeletal malocclusions: a cephalometric analysis. Progress in Orthodontics. 2015;16(1). | Excluded; no CBCT |
| 179 | Spin-Neto R, Hauge Matzen L, Hermann L, Fuglsig J, Wenzel A. Head motion and perception of discomfort by young children during simulated CBCT examinations. Dentomaxillofac Radiol. 2021;50(3):20200445. | Excluded; no CBCT |
| 180 | Stervik C, Lith A, Westerlund A, Ekestubbe A. Choice of radiography in orthodontic treatment on children and adolescents: A questionnaire-based study performed in Sweden. Eur J Oral Sci. 2021;129(4):e12796-e. | Excluded; no CBCT |
| 181 | Talaat WM, Adel OI, Al Bayatti S. Prevalence of temporomandibular disorders discovered incidentally during routine dental examination using the Research Diagnostic Criteria for Temporomandibular Disorders. Oral Surg Oral Med Oral Pathol Oral Radiol. 2018;125(3):250-9. | Excluded; no CBCT |
| 182 | Zhang Y, Yang ZG, Yang MX, Shi K, Li R, Diao KY, et al. Common atrium and the associated malformations Evaluation by low-dose dual-source computed tomography. Medicine (United States). 2018;97(46). | Excluded; no CBCT |
| 183 | {Nct} The Impact of Surgical Technique on PDC. https://clinicaltrialsgov/show/NCT02186548. 2014. | Excluded; no incidental findings sought |
| 184 | Adisen MZ, Misirlioglu M, Yorubulut S, Nalcaci R. Correlation of upper airway radiographic measurements with risk status for obstructive sleep apnea syndrome in young dental patients. Oral Surg Oral Med Oral Pathol Oral Radiol. 2017;123(1):129-36.e3. | Excluded; no incidental findings sought |
| 185 | Ahmed F, Brooks SL, Kapila SD. Efficacy of identifying maxillofacial lesions in cone-beam computed tomographs by orthodontists and orthodontic residents with third-party software. Am J Orthod Dentofacial Orthop. 2012;141(4):451-9. | Excluded; no incidental findings sought |
| 186 | Akyalcin S, Dyer DJ, English JD, Sar C. Comparison of 3-dimensional dental models from different sources: diagnostic accuracy and surface registration analysis. Am J Orthod Dentofacial Orthop. 2013;144(6):831-7. | Excluded; no incidental findings sought |
| 187 | Al-Gumaei WS, Al-Attab R, Alhammadi MS, Al-Rokhami RK, Almashraqi AA, Zhenlin G, et al. Evaluation of Spheno-occipital Synchondrosis Fusion in Chinese Population Using CBCT: A Cross-sectional Study. J Contemp Dent Pract. 2022;23(1):8-13. | Excluded; no incidental findings sought |
| 188 | Ali AH, Koller G, Foschi F, Andiappan M, Bruce KD, Banerjee A, et al. Self-Limiting versus Conventional Caries Removal: A Randomized Clinical Trial. Journal of Dental Research. 2018;97(11):1207-13. | Excluded; no incidental findings sought |
| 189 | Almeida MR, Futagami C, Conti AC, Oltramari-Navarro PV, Navarro Re. Dentoalveolar mandibular changes with self-ligating versus conventional bracket systems: A CBCT and dental cast study. Dental press journal of orthodontics. 2015;20(3):50-7. | Excluded; no incidental findings sought |
| 190 | Altieri F, Cassetta M. Comparison of changes in skeletal, dentoalveolar, periodontal, and nasal structures after tooth-borne or bone-borne rapid maxillary expansion: A parallel cohort study. AJO-DO. 2022;161(4):e336‐e44. | Excluded; no incidental findings sought |
| 191 | Anandarajah S, Abdalla Y, Dudhia R, Sonnesen L. Proposal of new upper airway margins in children assessed by CBCT. Dentomaxillofac Radiol. 2015;44(7):20140438. | Excluded; no incidental findings sought |
| 192 | Aziz T, Wheatley FC, Ansari K, Lagravere M, Major M, Flores-Mir C. Nasal septum changes in adolescent patients treated with rapid maxillary expansion. Dental Press J Orthod. 2016;21(1):47-53. | Excluded; no incidental findings sought |
| 193 | Bahrampour E, Zamani A, Kashkouli S, Soltanimehr E, Ghofrani Jahromi M, Sanaeian Pourshirazi Z. Accuracy of software designed for automated localization of the inferior alveolar nerve canal on cone beam CT images. Dento maxillo facial radiology. 2016;45(2):20150298. | Excluded; no incidental findings sought |
| 194 | Barbosa NMV, Castro AC, Conti F, Capelozza-Filho L, Almeida-Pedrin RR, Cardoso MA. Reliability and reproducibility of the method of assessment of midpalatal suture maturation: a tomographic study. Angle orthodontist. 2019;89(1):71‐7. | Excluded; no incidental findings sought |
| 195 | Becker K, Unland J, Wilmes B, Tarraf NE, Drescher D. Is there an ideal insertion angle and position for orthodontic mini-implants in the anterior palate? A CBCT study in humans. American Journal of Orthodontics and Dentofacial Orthopedics. 2019;156(3):345-54. | Excluded; no incidental findings sought |
| 196 | Bertram A, Eckert AW, Kolk A, Emshoff R. Panoramic prediction equations to estimate implant- to-mandibular canal dimensions in the mandibular posterior region: implications for dental implant treatment. Head Face Med. 2021;17(1):19-. | Excluded; no incidental findings sought |
| 197 | Botticelli S, Verna C, Cattaneo PM, Heidmann J, Melsen B. Two- versus three-dimensional imaging in subjects with unerupted maxillary canines. Eur J Orthod. 2011;33(4):344-9. | Excluded; no incidental findings sought |
| 198 | Brunetto M, Andriani Jda S, Ribeiro GL, Locks A, Correa M, Correa LR. Three-dimensional assessment of buccal alveolar bone after rapid and slow maxillary expansion: a clinical trial study. Am J Orthod Dentofacial Orthop. 2013;143(5):633-44. | Excluded; no incidental findings sought |
| 199 | Cantekin K, Sekerci AE, Miloglu O, Buyuk SK. Identification of the mandibular landmarks in a pediatric population. Med Oral Patol Oral Cir Bucal. 2014;19(2):e136-41. | Excluded; no incidental findings sought |
| 200 | Carlson ED, Stubbs A, Chandrasekaran S, Robles B, Kolanda R, Gohel A, et al. Sex-related differences in the distance of the mental foramen to the cementoenamel junction in adults: a study using cone beam computed tomography. General Dentistry. 2019;67(2):34-7. | Excluded; no incidental findings sought |
| 201 | Castro Júnior RCd. Avaliação comparativa da radiografia panorâmica e da tomografia computadorizada de feixe cônico prévio a extração de terceiros molares inferiores. 2016. p. 103-. | Excluded; no incidental findings sought |
| 202 | Celenk-Koca T, Erdinc AE, Hazar S, Harris L, English JD, Akyalcin S. Evaluation of miniscrew-supported rapid maxillary expansion in adolescents: A prospective randomized clinical trial. Angle Orthod. 2018;88(6):702-9. | Excluded; no incidental findings sought |
| 203 | Cheung GC, Dalci O, Mustac S, Papageorgiou SN, Hammond S, Darendeliler MA, et al. The upper airway volume effects produced by Hyrax, Hybrid-Hyrax, and Keles keyless expanders: a single-centre randomized controlled trial. European journal of orthodontics. 2021;43(3):254‐64. | Excluded; no incidental findings sought |
| 204 | Chun JH, de Castro ACR, Oh S, Kim KH, Choi SH, Nojima LI, et al. Skeletal and alveolar changes in conventional rapid palatal expansion (RPE) and miniscrew-assisted RPE (MARPE): a prospective randomized clinical trial using low-dose CBCT. BMC Oral Health. 2022;22(1):114. | Excluded; no incidental findings sought |
| 205 | Damstra J, Fourie Z, Huddleston Slater JJR, Ren Y. Reliability and the smallest detectable difference of measurements on 3-dimensional cone-beam computed tomography images. Am J Orthod Dentofacial Orthop. 2011;140(3):e107-14. | Excluded; no incidental findings sought |
| 206 | de Almeida MR, Butzke Marcal AS, Freire Fernandes TM, Vasconcelos JB, de Almeida RR, Nanda R. A comparative study of the effect of the intrusion arch and straight wire mechanics on incisor root resorption: A randomized, controlled trial. Angle Orthodontist. 2018;88(1):20-6. | Excluded; no incidental findings sought |
| 207 | de Castro Rizzi-Maia C, Maia-Filho EM, Nelson-Filho P, Segato RA, de Queiroz AM, Paula-Silva FW, et al. Single vs Two-session Root Canal Treatment: a Preliminary Randomized Clinical Study using Cone Beam Computed Tomography. Journal of contemporary dental practice. 2016;17(7):515‐21. | Excluded; no incidental findings sought |
| 208 | de Oliveira AEF, Cevidanes LHS, Phillips C, Motta A, Burke B, Tyndall D. Observer reliability of three-dimensional cephalometric landmark identification on cone-beam computerized tomography. Oral Surg Oral Med Oral Pathol Oral Radiol Endod. 2009;107(2):256-65. | Excluded; no incidental findings sought |
| 209 | Dunbar AC, Bearn D, McIntyre G. The influence of using digital diagnostic information on orthodontic treatment planning - a pilot study. J Healthc Eng. 2014;5(4):411-27. | Excluded; no incidental findings sought |
| 210 | Edwards R, Alsufyani N, Heo G, Flores-Mir C. Agreement among orthodontists experienced with cone-beam computed tomography on the need for follow-up and the clinical impact of craniofacial findings from multiplanar and 3-dimensional reconstructed views. Am J Orthod Dentofacial Orthop. 2015;148(2):264-73. | Excluded; no incidental findings sought |
| 211 | Elkordy SA, Abouelezz AM, Fayed MM, Attia KH, Ishaq RA, Mostafa YA. Three-dimensional effects of the mini-implant-anchored Forsus Fatigue Resistant Device: a randomized controlled trial. Angle orthodontist. 2016;86(2):292‐305. | Excluded; no incidental findings sought |
| 212 | ElSheshtawy AS, Nazzal H, El Shahawy OI, El Baz AA, Ismail SM, Kang J, et al. The effect of platelet-rich plasma as a scaffold in regeneration/revitalization endodontics of immature permanent teeth assessed using 2-dimensional radiographs and cone beam computed tomography: a randomized controlled trial. International endodontic journal. 2020;53(7):905-21. | Excluded; no incidental findings sought |
| 213 | Farronato G, Salvadori S, Nolet F, Zoia A, Farronato D. Assessment of inter- and intra-operator cephalometric tracings on cone beam ct radiographs: Comparison of the precision of the cone beam ct versus the latero-lateral radiograph tracing. Progress in Orthodontics. 2014;15(1). | Excluded; no incidental findings sought |
| 214 | Ferraro-Bezerra M, Tavares RN, de Medeiros JR, Nogueira AS, Avelar RL, Studart Soares EC. Effects of Pterygomaxillary Separation on Skeletal and Dental Changes After Surgically Assisted Rapid Maxillary Expansion: A Single-Center, Double-Blind, Randomized Clinical Trial. Journal of Oral and Maxillofacial Surgery. 2018;76(4):844-53. | Excluded; no incidental findings sought |
| 215 | Franco A, Ferreira Orestes SG, Coimbra EdF, Thevissen P, Fernandes A. Comparing dental identifier charting in cone beam computed tomography scans and panoramic radiographs using INTERPOL coding for human identification. Forensic Science International. 2019;302. | Excluded; no incidental findings sought |
| 216 | Gao Y, Lin Z, Rodella LF, Buffoli B, Wu X, Zhou Y. Piezoelectric ultrasonic bone surgery system in the extraction surgery of supernumerary teeth. Journal of cranio-maxillo-facial surgery. 2014;42(8):1577‐82. | Excluded; no incidental findings sought |
| 217 | Garib D, Miranda F, Palomo JM, Pugliese F, da Cunha Bastos JC, Dos Santos AM, et al. Orthopedic outcomes of hybrid and conventional Hyrax expanders. Angle orthodontist. 2021;91(2):178‐86. | Excluded; no incidental findings sought |
| 218 | Gerlach NL, Meijer GJ, Maal TJJ, Mulder J, Rangel FA, Borstlap WA, et al. Reproducibility of 3 different tracing methods based on cone beam computed tomography in determining the anatomical position of the mandibular canal. J Oral Maxillofac Surg. 2010;68(4):811-7. | Excluded; no incidental findings sought |
| 219 | Gunyuz Toklu M, Germec-Cakan D, Tozlu M. Periodontal, dentoalveolar, and skeletal effects of tooth-borne and tooth-bone-borne expansion appliances. Am J Orthod Dentofacial Orthop. 2015;148(1):97-109. | Excluded; no incidental findings sought |
| 220 | Hashem D, Mannocci F, Patel S, Manoharan A, Brown JE, Watson TF, et al. Clinical and Radiographic Assessment of the Efficacy of Calcium Silicate Indirect Pulp Capping: A Randomized Controlled Clinical Trial. Journal of Dental Research. 2015;94(4):562-8. | Excluded; no incidental findings sought |
| 221 | Hidalgo-Rivas JA, Theodorakou C, Carmichael F, Murray B, Payne M, Horner K. Use of cone beam CT in children and young people in three United Kingdom dental hospitals. Int J Paediatr Dent. 2014;24(5):336-48. | Excluded; no incidental findings sought |
| 222 | Holanda Ferreira FN, Gondim JO, Siebra Moreira Neto JJ, Fernandes dos Santos PC, de Freitas Pontes KM, Kurita LM, et al. Effects of low-level laser therapy on bone regeneration of the midpalatal suture after rapid maxillary expansion. Lasers in Medical Science. 2016;31(5):907-13. | Excluded; no incidental findings sought |
| 223 | Husson AH, Burhan AS, Hajeer MY, Nawaya FR. Evaluation of the dimensional changes in the mandible, condyles, and the temporomandibular joint following skeletal class III treatment with chin cup and bonded maxillary bite block using low-dose computed tomography: A single-center, randomized controlled trial. F1000Research. 2023;12. | Excluded; no incidental findings sought |
| 224 | Ilgüy D, Ilgüy M, Fisekçioglu E, Dölekoglu S, Ersan N. Articular eminence inclination, height, and condyle morphology on cone beam computed tomography. ScientificWorldJournal. 2014;2014:761714-. | Excluded; no incidental findings sought |
| 225 | Kabalan O, Gordon J, Heo G, Lagravère MO. Nasal airway changes in bone-borne and tooth-borne rapid maxillary expansion treatments. International orthodontics. 2015;13(1):1‐15. | Excluded; no incidental findings sought |
| 226 | Kalra S, Tripathi T, Rai P, Kanase A. Evaluation of orthodontic mini-implant placement: a CBCT study. Progress in orthodontics. 2014;15:61. | Excluded; no incidental findings sought |
| 227 | Lagravère MO, Gamble J, Major PW, Heo G. Transverse dental changes after tooth-borne and bone-borne maxillary expansion. International orthodontics. 2013;11(1):21‐34. | Excluded; no incidental findings sought |
| 228 | Lagravère MO, Low C, Flores-Mir C, Chung R, Carey JP, Heo G, et al. Intraexaminer and interexaminer reliabilities of landmark identification on digitized lateral cephalograms and formatted 3-dimensional cone-beam computerized tomography images. Am J Orthod Dentofacial Orthop. 2010;137(5):598-604. | Excluded; no incidental findings sought |
| 229 | Lahoud P, EzEldeen M, Beznik T, Willems H, Leite A, Van Gerven A, et al. Artificial Intelligence for Fast and Accurate 3-Dimensional Tooth Segmentation on Cone-beam Computed Tomography. Journal of endodontics. 2021;47(5):827-35. | Excluded; no incidental findings sought |
| 230 | Lei J, Yap AU, Liu MQ, Fu KY. Condylar repair and regeneration in adolescents/young adults with early-stage degenerative temporomandibular joint disease: A randomised controlled study. J Oral Rehabil. 2019;46(8):704-14. | Excluded; no incidental findings sought |
| 231 | Leite V, Conti AC, Navarro R, Almeida M, Oltramari-Navarro P, Almeida R. Comparison of root resorption between self-ligating and conventional preadjusted brackets using cone beam computed tomography. Angle Orthodontist. 2012;82(6):1078-82. | Excluded; no incidental findings sought |
| 232 | Liang YH, Jiang LM, Jiang L, Chen XB, Liu YY, Tian FC, et al. Radiographic healing after a root canal treatment performed in single-rooted teeth with and without ultrasonic activation of the irrigant: a randomized controlled trial. Journal of endodontics. 2013;39(10):1218‐25. | Excluded; no incidental findings sought |
| 233 | Major MP, Witmans M, El-Hakim H, Major PW, Flores-Mir C. Agreement between cone-beam computed tomography and nasoendoscopy evaluations of adenoid hypertrophy. Am J Orthod Dentofacial Orthop. 2014;146(4):451-9. | Excluded; no incidental findings sought |
| 234 | Marcussen L, Stokbro K, Aagaard E, Torkov P, Thygesen T. Changes in Upper Airway Volume Following Orthognathic Surgery. J Craniofac Surg. 2017;28(1):66-70. | Excluded; no incidental findings sought |
| 235 | Massaro C, Garib D, Cevidanes L, Janson G, Yatabe M, Lauris JRP, et al. Maxillary dentoskeletal outcomes of the expander with differential opening and the fan-type expander: a randomized controlled trial. Clinical oral investigations. 2021;25(9):5247-56. | Excluded; no incidental findings sought |
| 236 | Matzen LH, Hintze H, Spin-Neto R, Wenzel A. Reproducibility of mandibular third molar assessment comparing two cone beam CT units in a matched pairs design. Dentomaxillofac Radiol. 2013;42(10):20130228-. | Excluded; no incidental findings sought |
| 237 | Matzen LH, Petersen LB, Wenzel A. Radiographic methods used before removal of mandibular third molars among randomly selected general dental clinics. Dento maxillo facial radiology. 2016;45(4):20150226. | Excluded; no incidental findings sought |
| 238 | Mehrdad L, Malekafzali B, Shekarchi F, Safi Y, Asgary S. Histological and CBCT evaluation of a pulpotomised primary molar using calcium enriched mixture cement. Eur Arch Paediatr Dent. 2013;14(3):191-4. | Excluded; no incidental findings sought |
| 239 | Migliorati M, Cevidanes L, Sinfonico G, Drago S, Dalessandri D, Isola G, et al. Three dimensional movement analysis of maxillary impacted canine using TADs: a pilot study. Head & face medicine. 2021;17(1):1. | Excluded; no incidental findings sought |
| 240 | Miranda F, Garib D, Pugliese F, da Cunha Bastos JC, Janson G, Palomo JM. Upper airway changes in Class III patients using miniscrew-anchored maxillary protraction with hybrid and hyrax expanders: a randomized controlled trial. Clinical oral investigations. 2022;26(1):183‐95. | Excluded; no incidental findings sought |
| 241 | Nair AK, Jose M, Sreela LS, Prasad TS, Mathew P. Prevalence and pattern of proximity of maxillary posterior teeth to maxillary sinus with mucosal thickening: A cone beam computed tomography based retrospective study. Annals of African Medicine. 2023;22(3):327-32. | Excluded; no incidental findings sought |
| 242 | Naji P, Alsufyani NA, Lagravère MO. Reliability of anatomic structures as landmarks in three-dimensional cephalometric analysis using CBCT. Angle Orthod. 2014;84(5):762-72. | Excluded; no incidental findings sought |
| 243 | Naoumova J, Kjellberg H, Palm R. Cone-beam computed tomography for assessment of palatal displaced canine position: a methodological study. Angle Orthod. 2014;84(3):459-66. | Excluded; no incidental findings sought |
| 244 | Naoumova J, Kurol J, Kjellberg H. Extraction of the deciduous canine as an interceptive treatment in children with palatal displaced canines - part I: shall we extract the deciduous canine or not? Eur J Orthod. 2015;37(2):209-18. | Excluded; no incidental findings sought |
| 245 | Nikolaev AE, Kadieva AS, Shapiev AN, Chernina VY, Artsybasheva MV, Gonchar AP, et al. X-ray diagnostics of dentoalveolar disorders in children and adolescents. Voprosy Prakticheskoi Pediatrii. 2019;14(2):43-54. | Excluded; no incidental findings sought |
| 246 | Nogueira AS. Avaliação da prevalência de variações anatômicas do complexo ostiomeatal e de afecções inflamatórias dos seios maxilares por meio da tomografia computadorizada de feixe cônico. 2013. p. 130-. | Excluded; no incidental findings sought |
| 247 | Nowzari H, Molayem S, Chiu CHK, Rich SK. Cone Beam Computed Tomographic Measurement of Maxillary Central Incisors to Determine Prevalence of Facial Alveolar Bone Width >= 2 mm. Clinical Implant Dentistry and Related Research. 2012;14(4):595-602. | Excluded; no incidental findings sought |
| 248 | Nucera R, Costa S, Bellocchio AM, Barbera S, Drago S, Silvestrini A, et al. Evaluation of palatal bone depth, cortical bone, and mucosa thickness for optimal orthodontic miniscrew placement performed according to the third palatal ruga clinical reference. European Journal of Orthodontics. 2022. | Excluded; no incidental findings sought |
| 249 | Oenning AC, Pauwels R, Stratis A, De Faria Vasconcelos K, Tijskens E, De Grauwe A, et al. Halve the dose while maintaining image quality in paediatric Cone Beam CT. Sci Rep. 2019;9(1):5521. | Excluded; no incidental findings sought |
| 250 | Orhan K, Gorurgoz C, Akyol M, Ozarslanturk S, Avsever H. An anatomical variant: evaluation of accessory canals of the canalis sinuosus using cone beam computed tomography. Folia Morphologica. 2018;77(3):551-7. | Excluded; no incidental findings sought |
| 251 | Pachêco-Pereira C, Alsufyani NA, Major M, Heo G, Flores-Mir C. Accuracy and reliability of orthodontists using cone-beam computerized tomography for assessment of adenoid hypertrophy. Am J Orthod Dentofacial Orthop. 2016;150(5):782-8. | Excluded; no incidental findings sought |
| 252 | Pachêco-Pereira C, Alsufyani NA, Major MP, Flores-Mir C. Accuracy and reliability of oral maxillofacial radiologists when evaluating cone-beam computed tomography imaging for adenoid hypertrophy screening: a comparison with nasopharyngoscopy. Oral Surg Oral Med Oral Pathol Oral Radiol. 2016;121(6):e168-74. | Excluded; no incidental findings sought |
| 253 | Palone M, Panzeri P, Cremonini F, Spedicato GA, Squarci V, Albertini P. Effect of conventional versus passive self-ligating vestibular appliances on torque, tip and transverse dental changes in patients affected by class i malocclusion: A retrospective study. Pesquisa Brasileira em Odontopediatria e Clinica Integrada. 2021;21. | Excluded; no incidental findings sought |
| 254 | Prasanna Arvind TR, Ramasamy N, Subramanian AK, Selvaraj A, Siva S. Three-dimensional volumetric evaluation of root resorption in maxillary anteriors following en-masse retraction with varying force vectors - a randomized control trial. Orthodontics & craniofacial research. 2023. | Excluded; no incidental findings sought |
| 255 | Romulo de Medeiros J, Ferraro Bezerra M, Gurgel Costa FW, Pinheiro Bezerra T, de Araajo Alencar CR, Studart Soares EC. Does pterygomaxillary disjunction in surgically assisted rapid maxillary expansion influence upper airway volume? A prospective study using Dolphin Imaging 3D. International Journal of Oral and Maxillofacial Surgery. 2017;46(9):1094-101. | Excluded; no incidental findings sought |
| 256 | Różyło-Kalinowska I, Kalinowski P, Krasicka E, Galić I, Mehdi F, Cameriere R. The Cameriere method using cone-beam computed tomography (CBCT) scans for dental age estimation in children. Australian Journal of Forensic Sciences. 2020:1-15. | Excluded; no incidental findings sought |
| 257 | Ruan M-J, Chen G, Xu T-M. Comparison of orthodontic tooth movement between adolescents and adults based on implant superimposition. Plos One. 2018;13(5). | Excluded; no incidental findings sought |
| 258 | Sampermans G, Sawaljanow A, Proff P, Kirschneck C, Paddenberg E. Ideal transverse position of mandibular first molars based on CBCT-derived alveolar bone coverage. Ann Anat. 2022;241:151908-. | Excluded; no incidental findings sought |
| 259 | Shahidi S, Bahrampour E, Soltanimehr E, Zamani A, Oshagh M, Moattari M, et al. The accuracy of a designed software for automated localization of craniofacial landmarks on CBCT images. BMC Med Imaging. 2014;14:32-. | Excluded; no incidental findings sought |
| 260 | Sirri MR, Burhan AS, Hajeer MY, Nawaya FR. Evaluation of corticision-based acceleration of lower anterior teeth alignment in terms of root resorption and dehiscence formation using cone-beam computed tomography in young adult patients: A randomized controlled trial. International orthodontics. 2021;19(4):580-90. | Excluded; no incidental findings sought |
| 261 | Suzuki M, Deguchi T, Watanabe H, Seiryu M, Iikubo M, Sasano T, et al. Evaluation of optimal length and insertion torque for miniscrews. American Journal of Orthodontics and Dentofacial Orthopedics. 2013;144(2):251-9. | Excluded; no incidental findings sought |
| 262 | Tai B, Goonewardene MS, Murray K, Koong B, Islam SMS. The reliability of using postero-anterior cephalometry and cone-beam CT to determine transverse dimensions in clinical practice. Aust Orthod J. 2014;30(2):132-42. | Excluded; no incidental findings sought |
| 263 | Tai K, Hotokezaka H, Park JH, Tai H, Miyajima K, Choi M, et al. Preliminary cone-beam computed tomography study evaluating dental and skeletal changes after treatment with a mandibular Schwarz appliance. American Journal of Orthodontics and Dentofacial Orthopedics. 2010;138(3):262.e1-.e11. | Excluded; no incidental findings sought |
| 264 | Tai K, Park JH, Mishima K, Shin J-W. 3-Dimensional cone-beam computed tomography analysis of transverse changes with Schwarz appliances on both jaws. Angle Orthodontist. 2011;81(4):670-7. | Excluded; no incidental findings sought |
| 265 | Tai K, Park JH. Dental and skeletal changes in the upper and lower jaws after treatment with Schwarz appliances using cone-beam computed tomography. Journal of clinical pediatric dentistry. 2010;35(1):111‐20. | Excluded; no incidental findings sought |
| 266 | Tang M, Guo H-m, Bai Y-x, Li S. [Application of integrated digital maxillodental model in computer aided design of individualized lingual brackets]. Zhonghua kou qiang yi xue za zhi = Zhonghua kouqiang yixue zazhi = Chinese journal of stomatology. 2012;47(8):501-4. | Excluded; no incidental findings sought |
| 267 | Titiz I, Laubinger M, Keller T, Hertrich K, Hirschfelder U. Repeatability and reproducibility of landmarks-a three-dimensional computed tomography study. European Journal of Orthodontics. 2012;34(3):276-86. | Excluded; no incidental findings sought |
| 268 | Torun GS. Soft tissue changes in the orofacial region after rapid maxillary expansion : A cone beam computed tomography study. J Orofac Orthop. 2017;78(3):193-200. | Excluded; no incidental findings sought |
| 269 | Tsukiboshi Y, Tanikawa C, Yamashiro T. Surface-based 3-dimensional cephalometry: An objective analysis of cranio-mandibular morphology. American Journal of Orthodontics and Dentofacial Orthopedics. 2020;158(4):535-46. | Excluded; no incidental findings sought |
| 270 | Van Gorp G, Lambrechts M, Jacobs R, Declerck D. Does clinical experience with dental traumatology impact 2D and 3D radiodiagnostic performance in paediatric dentists? An exploratory study. BMC Oral Health. 2022;22(1):245. | Excluded; no incidental findings sought |
| 271 | Van Gorp G, Lambrechts M, Jacobs R, Declerck D. Paediatric dentist's ability to detect and diagnose dental trauma using 2D versus 3D imaging. Eur Arch Paediatr Dent. 2021;22(4):699-705. | Excluded; no incidental findings sought |
| 272 | Wang R-y, Han M, Liu H, Wang C-l, Xian H-h, Zhang L, et al. Establishment of reference mandibular plane for anterior alveolar morphology evaluation using cone beam computed tomography. J Zhejiang Univ Sci B. 2012;13(11):942-7. | Excluded; no incidental findings sought |
| 273 | Wu D, Hu D-q, Xin B-c, Sun D-g, Ge Z-p, Su J-y. Root canal morphology of maxillary and mandibular first premolars analyzed using cone-beam computed tomography in a Shandong Chinese population. Medicine. 2020;99(20). | Excluded; no incidental findings sought |
| 274 | Zamora N, Llamas J-M, Cibrián R, Gandia J-L, Paredes V. A study on the reproducibility of cephalometric landmarks when undertaking a three-dimensional (3D) cephalometric analysis. Med Oral Patol Oral Cir Bucal. 2012;17(4):e678-88. | Excluded; no incidental findings sought |
| 275 | Zandi M, Miresmaeili A, Heidari A. Short-term skeletal and dental changes following bone-borne versus tooth-borne surgically assisted rapid maxillary expansion: a randomized clinical trial study. Journal of cranio-maxillo-facial surgery. 2014;42(7):1190‐5. | Excluded; no incidental findings sought |
| 276 | Zeitounlouian TS, Zeno KG, Brad BA, Haddad RA. Three-dimensional evaluation of the effects of injectable platelet rich fibrin (i-PRF) on alveolar bone and root length during orthodontic treatment: a randomized split mouth trial. BMC oral health. 2021;21(1):92. | Excluded; no incidental findings sought |
| 277 | Zekry A, Wang R, Chau ACM, Lang NP. Facial alveolar bone wall width - a cone-beam computed tomography study in Asians. Clinical Oral Implants Research. 2014;25(2):194-206. | Excluded; no incidental findings sought |
| 278 | Zhang CX, Shen G, Ning YJ, Liu H, Zhao Y, Liu DX. Effects of Twin-block vs sagittal-guidance Twin-block appliance on alveolar bone around mandibular incisors in growing patients with Class II Division 1 malocclusion. Am J Orthod Dentofacial Orthop. 2020;157(3):329-39. | Excluded; no incidental findings sought |
| 279 | Alsufyani N. Incidental cone beam CT finding of juvenile pleomorphic adenoma. Case Reports in Dentistry. 2020;2020. | Excluded; case report / series |
| 280 | Barghan S, Tetradis S, Nervina JM. Skeletal and soft-tissue incidental findings on cone-beam computed tomography images. Am J Orthod Dentofacial Orthop. 2013;143(6):888-92. | Excluded; case report / series |
| 281 | Cossellu G, Angiero F, Farronato G. Complete pre-eruptive idiopathic crown resorption. Pediatr Dent. 2014;36(7):147-50. | Excluded; case report / series |
| 282 | Demirturk Kocasarac H, Celenk P, Erzurumlu Z, Kutlar G. Clinical and radiological aspects of rhinoliths: report of five cases. Oral Surg Oral Med Oral Pathol Oral Radiol. 2013;116(2):232-7. | Excluded; case report / series |
| 283 | Hansel D, Irala LED. Reabsorção externa cervical: relato de caso clínico. Stomatos. 2014;20(38):51-9. | Excluded; case report / series |
| 284 | Helal N, Basri O, Gadi LS, Alhameed AF, Grady JM. Parents' Perceptions of Breathing Pattern Changes, Sleep Quality, and Fatigue in Children after Rapid Maxillary Expansion: A Survey and Case Series Study. Open Dentistry Journal. 2019;13:85-92. | Excluded; case report / series |
| 285 | Khalifa HM, Barayan MA. Ossification of Pterygospinous Ligament: An Incidental Finding in Cone Beam Computed Tomography. Am J Case Rep. 2021;22:e928061. | Excluded; case report / series |
| 286 | Kulczyk T, Daktera-Micker A, Biedziak B, Wziatek A, Bednarek-Rajewska K. The primary outbreaks of burkitt lymphoma in the oral cavity. A report of two cases, review of the literature and dental implications. Diagnostics. 2021;11(12). | Excluded; case report / series |
| 287 | Leven AJ, Sood B. Pathosis or Additional Maxillary Neurovascular Channel? A Case Report. J Endod. 2018;44(6):1048-51. | Excluded; case report / series |
| 288 | Lyros I, Fora E, Damaskos S, Stanko P, Tsolakis A. An incidental finding on a diagnostic CBCT: a case report. Aust Orthod J. 2014;30(1):67-71. | Excluded; case report / series |
| 289 | Manmontri C, Mahasantipiya PM, Chompu-inwai P. Preeruptive Intracoronal Radiolucencies: Detection and Nine Years Monitoring with a Series of Dental Radiographs. Case Reports in Dentistry. 2017;2017. | Excluded; case report / series |
| 290 | Newaz ZA, Barghan S, Katkar RA, Bennett JA, Nair MK. Incidental findings of skull-base abnormalities in cone-beam computed tomography scans with consultation by maxillofacial radiologists. Am J Orthod Dentofacial Orthop. 2015;147(1):127-31. | Excluded; case report / series |
| 291 | Oenning AC, Oliveira LB, Junqueira JLC, Sousa Melo SL. Buccal bifurcation cyst as an incidental finding in cone beam computed tomography scans tomografia computadorizada de feixe cônico. RGO - Revista Gaúcha de Odontologia. 2018;66(4):385-9. | Excluded; case report / series |
| 292 | Parker K, Visram S, Hodges S. An incidental finding of a long-standing button battery in the floor of the nose during a routine orthodontic examination. J Orthod. 2016;43(2):147-50. | Excluded; case report / series |
| 293 | Popat H, Drage N, Durning P. Mid-line clefts of the cervical vertebrae - an incidental finding arising from cone beam computed tomography of the dental patient. Br Dent J. 2008;204(6):303-6. | Excluded; case report / series |
| 294 | Rogers SA, Drage N, Durning P. Incidental findings arising with cone beam computed tomography imaging of the orthodontic patient. Angle Orthodontist. 2011;81(2):350-5. | Excluded; case report / series |
| 295 | Sane VD, Chandan S, Patil S, Patil K. Cone Beam Computed Tomography Heralding New Vistas in Appropriate Diagnosis and Efficient Management of Incidentally Found Impacted Mesiodens. Journal of Craniofacial Surgery. 2017;28(2):e105-e6. | Excluded; case report / series |
| 296 | Sezgin OS, Kayipmaz S. Trifid mandibular condyle. Oral Radiology. 2009;25(2):146-8. | Excluded; case report / series |
| 297 | Yan DJ, Lenoir V, Chatelain S, Stefanelli S, Becker M. Congenital vomer agenesis: A rare and poorly understood condition revealed by cone beam CT. Diagnostics. 2018;8(1). | Excluded; case report / series |
| 298 | Alsufyani NA. Cone beam computed tomography incidental findings of the cervical spine and clivus: retrospective analysis and review of the literature. Oral Surgery Oral Medicine Oral Pathology Oral Radiology. 2017;123(6):E197-E217. | Mixed sample; received data; included |
| 299 | Bayrakdar IS, Miloglu O, Altun O, Gumussoy I, Durna D, Yilmaz AB. Cone beam computed tomography imaging of ponticulus posticus: Prevalence, characteristics, and a review of the literature. Oral Surgery, Oral Medicine, Oral Pathology and Oral Radiology. 2014;118(6):e210-e9. | Included |
| 300 | Cantekin K, Şekerci A. Evaluation of the accessory mental foramen in a pediatric population using cone-beam computed tomography. J Clin Pediatr Dent. 2014;39(1):85-9. | Included |
| 301 | Cobb Jr. JW. Pathology observed on cone beam computed tomographic scans: a comparison of prevalence and type of incidental findings for child/adolescents and adults. MSc Thesis, 2013, San Antonio. | Included |
| 302 | Dogramaci EJ, Rossi-Fedele G, McDonald F. Clinical importance of incidental findings reported on small-volume dental cone beam computed tomography scans focused on impacted maxillary canine teeth. Oral Surg Oral Med Oral Pathol Oral Radiol. 2014;118(6):e205-9. | Mixed sample; received data; included |
| 303 | Geist JR, Geist SM, Lin LM. A cone beam CT investigation of ponticulus posticus and lateralis in children and adolescents. Dentomaxillofac Radiol. 2014;43(5):20130451. | Included |
| 304 | Giaccaglia F, Bruno G, Gracco A, De Stefani A. Incidental findings of the nose and paranasal sinuses in orthodontic patients in the age of development: a retrospective study on CBCTs. Eur J Paediatr Dent. 2022 Sep;23(3):189-193. | Included |
| 305 | Etemad L, Mehta S, Lurie AG, Tadinada A. Prevalence and Clinical Significance of Incidental Findings in the Maxillofacial Complex of Adolescent Orthodontic Patients: A Retrospective Cone Beam Computed Tomography Analysis. Cureus. 2023;15(10):e47480. https://doi. org/10.7759/cureus.47480. | Included |
| 306 | Lopes IA, Tucunduva RM, Handem RH, Capelozza AL. Study of the frequency and location of incidental findings of the maxillofacial region in different fields of view in CBCT scans. Dento maxillo facial radiology. 2017;46(1):20160215. | Mixed sample; received data; included |
| 307 | Lopes IA. Estudo da frequência e localização de achados incidentais da região maxilofacial de diferentes campos de visão em exames de tomografia computadorizada de feixe cônico. 2016. p. 136-. | Mixed sample; received data; included |
| 308 | Togan B, Gander T, Lanzer M, Martin R, Lübbers H-T. Incidence and frequency of nondental incidental findings on cone-beam computed tomography. J Craniomaxillofac Surg. 2016;44(9):1373-80. | Mixed sample; received data; included |

CBCT, cone beam computed tomography.

**Supplementary Table 5**. Communication attempts to authors of identified studies with their current status.

| **Nr** | **Paper** | **Contact reason** | **Communication history** | **Status** |
| --- | --- | --- | --- | --- |
| 1 | Alsufyani NA. Cone beam computed tomography incidental findings of the cervical spine and clivus: retrospective analysis and review of the literature. Oral Surgery Oral Medicine Oral Pathology Oral Radiology. 2017;123(6):E197-E217. | Separate for children | Data sent | Ultimately included |
| 2 | Dogramaci EJ, Rossi-Fedele G, McDonald F. Clinical importance of incidental findings reported on small-volume dental cone beam computed tomography scans focused on impacted maxillary canine teeth. Oral Surg Oral Med Oral Pathol Oral Radiol. 2014;118(6):e205-9. | Separate for children | Data sent | Ultimately included |
| 3 | Lopes IA, Tucunduva RM, Handem RH, Capelozza AL. Study of the frequency and location of incidental findings of the maxillofacial region in different fields of view in CBCT scans. Dento maxillo facial radiology. 2017;46(1):20160215. | Separate for children | Data sent | Ultimately included |
| 4 | Lopes IA. Estudo da frequência e localização de achados incidentais da região maxilofacial de diferentes campos de visão em exames de tomografia computadorizada de feixe cônico. 2016. p. 136-. | Separate for children |  | Ultimately included |
| 5 | Togan B, Gander T, Lanzer M, Martin R, Lübbers H-T. Incidence and frequency of nondental incidental findings on cone-beam computed tomography. J Craniomaxillofac Surg. 2016;44(9):1373-80. | Separate for children | Data sent | Ultimately included |
| 6 | Altindag A, Avsever H, Borahan O, Akyol M, Orhan K. Incidental Findings in Cone-Beam Computed Tomographic Images: Calcifications in Head and Neck Region. Balk J Dent Med, 2017; 100-107. | Separate for children | No answer | Ultimately excluded |
| 7 | Bayrak S, Bulut DG, Çakmak ESK, Orhan K. Cone beam computed tomographic evaluation of intracranial physiologic calcifications. Journal of Craniofacial Surgery. 2019;30(2):510-3. | Separate for children | No answer. | Ultimately excluded |
| 8 | Borghesi A, Michelini S, Zigliani A, Tonni I, Maroldi R. Three-rooted maxillary first premolars incidentally detected on cone beam CT: an in vivo study. Surgical and Radiologic Anatomy. 2019;41(4):461-8. | Separate for children | No answer | Ultimately excluded |
| 9 | Braun MJ, Rauneker T, Dreyhaupt J, Hoffmann TK, Luthardt RG, Schmitz B, et al. Dental and Maxillofacial Cone Beam CT—High Number of Incidental Findings and Their Impact on Follow-Up and Therapy Management. Diagnostics. 2022;12(5). | Separate for children | No answer | Ultimately excluded |
| 10 | Bruno G, Stefani AD, Benetazzo C, Cavallin F, Gracco A. Changes in nasal septum morphology after rapid maxillary expansion: a Cone-Beam Computed Tomography study in pre-pubertal patient. Dental Press J Orthod. 2020;25(5):51-6. | Number of CBCTs searched where the 20 incidental findings of nasal septum deviation was found. | No answer | Ultimately excluded |
| 11 | Çaǧlayan F, Tozoǧlu U. Incidental findings in the maxillofacial region detected by cone beam CT. Diagnostic and Interventional Radiology. 2012;18(2):159-63. | Separate for children | No answer | Ultimately excluded |
| 12 | Cha J-Y, Mah J, Sinclair P. Incidental findings in the maxillofacial area with 3-dimensional cone-beam imaging. American Journal of Orthodontics and Dentofacial Orthopedics. 2007;132(1):7-14. | Separate for children | No answer | Ultimately excluded |
| 13 | Choi J-Y, Oh SH, Kim S-H, Ahn H-W, Kang Y-G, Choi Y-S, et al. Effectiveness of 2D radiographs in detecting CBCT-based incidental findings in orthodontic patients. Sci Rep. 2021;11(1):9280-. | Separate for children | No answer | Ultimately excluded |
| 14 | Drage N, Rogers S, Greenall C, Playle R. Incidental findings on cone beam computed tomography in orthodontic patients. Journal of Orthodontics. 2013;40(1):29-37. | Separate for children | No answer | Ultimately excluded |
| 15 | Edwards R, Alsufyani N, Heo G, Flores-Mir C. The frequency and nature of incidental findings in large-field cone beam computed tomography scans of an orthodontic sample. Progress in orthodontics. 2014;15(1):37. | Separate for children | No answer | Ultimately excluded |
| 16 | Evirgen S, Yuksel HT, Yuksel G, Kaki B, Kamburoglu K. Assessment of intravertebral pneumatocysts, degenerative joint disease, and ponticulus posticus in the cervical spine through cone beam computed tomography examination. Oral Surgery Oral Medicine Oral Pathology Oral Radiology. 2020;129(5):531-8. | Separate for children | No answer | Ultimately excluded |
| 17 | Gümrü B, Guldali M, Tarcin B, Idman E, Sertac Peker M. Evaluation of cone beam computed tomography referral profile: Retrospective study in a Turkish paediatric subpopulation. Eur J Paediatr Dent. 2021;22(1):66-70. | Frequency of each separate incidental finding type, not overall. | Answered but did not send data | Ultimately excluded |
| 18 | Oliveira RdS, Peretto JT, Panzarella FK, Raitz R. Evaluation of Incidental Findings on Cone Beam Computed Tomography. Pesqui bras odontopediatria clín integr. 2019;19(1):4340-. | Separate for children | No answer | Ultimately excluded |
| 19 | Pazera P, Bornstein MM, Pazera A, Sendi P, Katsaros C. Incidental maxillary sinus findings in orthodontic patients: A radiographic analysis using cone-beam computed tomography (CBCT). Orthodontics and Craniofacial Research. 2011;14(1):17-24. | Separate for children | No answer | Ultimately excluded |
| 20 | Price JB, Thaw KL, Tyndall DA, Ludlow JB, Padilla RJ. Incidental findings from cone beam computed tomography of the maxillofacial region: a descriptive retrospective study. Clin Oral Implants Res. 2012;23(11):1261-8. | Separate for children | Answered but did not send data | Ultimately excluded |
| 21 | Thaw KL. Incidental findings from cone beam computed tomography of the maxillofacial region: a descriptive retrospective study. MSc Thesis, 2010, Chapel Hill. | Separate for children | Answered but did not send data | Ultimately excluded |
| 22 | Ritter L, Lutz J, Neugebauer J, Scheer M, Dreiseidler T, Zinser MJ, et al. Prevalence of pathologic findings in the maxillary sinus in cone-beam computerized tomography. Oral Surgery Oral Medicine Oral Pathology Oral Radiology and Endodontology. 2011;111(5):634-40. | Separate for children | No answer | Ultimately excluded |
| 23 | Shokri A, Mortazavi H, Baharvand M, Falah-Kooshki S, Ostovarrad F, Karimi A. Prevalence of incidental findings in paranasal sinuses using CBCT. Dental and Medical Problems. 2014;51(4):431-8. | Separate for children | No answer | Ultimately excluded |
| 24 | Theodoridis C, Vaitsidis Z, Angelopoulos C. Incidental findings on CBCT and classification according to their significance. Oral Surg Oral Med Oral Pathol Oral Radiol 2019;127(1):47. | Separate for children | Answered but did not send data | Ultimately excluded |
| 25 | Pliska B, DeRocher M, Larson BE. Incidence of significant findings on CBCT scans of an orthodontic patient population. Northwest Dent. 2011;90(2):12-6. | Separate for children | Answered but did not send data | Ultimately excluded |

X

**Supplementary Table 6.** Assessment of methodological soundness / internal validity / reporting completeness of included studies.

| **Question** | **Alsufyani 2017** | **Bayrakdar 2014** | **Cantekin 2014** | **Cobb 2013** | **Dogramaci 2014** | **Etemad 2023** | **Geist 2014** | **Giaccaglia 2022** | **Lopes 2016; 2017** | **Togan 2016** |
| --- | --- | --- | --- | --- | --- | --- | --- | --- | --- | --- |
| Are details given about the origin of the analyzed sample? | **Yes** | **Yes** | **Yes** | **Yes** | **Yes** | **Yes** | **Yes** | **Yes** | **Yes** | **Yes** |
| Is the acquisition timeframe of included CBCT images given? | **Yes** | **Yes** | **No** | **Yes** | **Yes** | **No** | **Yes** | **No** | **No** | **Yes** |
| According to eligibility criteria, are included patients representative of the general healthy population, the target population of interest? | **Unclear** | **Unclear** | **Yes** | **Yes** | **No** | **No** | **Unclear** | **No** | **Yes** | **Unclear** |
| Were included cases selected consecutively or randomly? | **Yes** | **Unclear** | **Yes** | **Yes** | **Unclear** | **Yes** | **Unclear** | **Unclear** | **Unclear** | **Unclear** |
| Is the age of included patients reported? | **Yes** | **Partly** | **Yes** | **Partly** | **Yes** | **Partly** | **Yes** | **Yes** | **Yes** | **Yes** |
| Is the sex of included patients reported? | **Yes** | **No** | **Yes** | **No** | **No** | **No** | **Yes** | **Yes** | **Yes** | **Yes** |
| Is the ethnicity of included patients reported? | **No** | **No** | **No** | **No** | **No** | **No** | **Yes** | **No** | **No** | **No** |
| Is the reason for acquisition of CBCT images reported? | **Yes** | **No** | **Yes** | **Yes** | **Yes** | **Yes** | **Yes** | **Yes** | **Yes** | **No** |
| Are inclusion / exclusion criteria of eligible patients reported? | **No** | **No** | **Yes** | **Partly** | **Partly** | **Yes** | **No** | **Yes** | **Yes** | **No** |
| According to the reason for CBCT acquisition, are included patients representative of the general population? | **Yes** | **Unclear** | **Yes** | **Yes** | **No** | **No** | **No** | **No** | **Yes** | **Unclear** |
| Do the authors state which area was included in the CBCT? | **Yes** | **Unclear** | **Unclear** | **No** | **Yes** | **Yes** | **Unclear** | **Yes** | **Yes** | **Yes** |
| Are technical details about the acquired CBCT image given? (FOV, voxel, time, etc) | **No** | **Partly** | **Partly** | **Yes** | **Partly** | **Yes** | **Yes** | **No** | **Partly** | **Partly** |
| Are CBCT images evaluated by an experienced certified radiologist? | **Yes** | **Yes** | **Unclear** | **Yes** | **Yes** | **Yes** | **Yes** | **Unclear** | **Yes** | **No** |
| Are CBCT assessors unaware of the reason for CBCT acquisition? | **Unclear** | **Unclear** | **Unclear** | **Yes** | **Unclear** | **Yes** | **Unclear** | **Unclear** | **Unclear** | **Unclear** |
| Have efforts been put into place to make sure optimal assessing conditions exist and errors are minimized? | **Unclear** | **Unclear** | **Unclear** | **Yes** | **Unclear** | **Yes** | **Unclear** | **Unclear** | **Partly** | **Unclear** |
| Were all CBCT images assessed under the same conditions? | **Unclear** | **Unclear** | **Unclear** | **Yes** | **Unclear** | **Yes** | **Unclear** | **Unclear** | **Unclear** | **Unclear** |
| Have CBCT images been assessed from at least 2 assessors independently? | **Unclear** | **Yes** | **Unclear** | **No** | **Unclear** | **Yes** | **Unclear** | **Unclear** | **Yes** | **Unclear** |
| Have findings been reported on both patient level and CBCT image level, if applicable (multiple findings per image clarification)? | **Yes** | **Yes** | **Partly** | **No** | **Yes** | **No** | **Yes** | **Yes** | **Yes** | **Yes** |
| In case categorizations / poolings of findings have been made, have also separate individual findings been reported? | **Yes** | **Yes** | **Yes** | **Yes** | **Yes** | **Yes** | **Yes** | **Yes** | **Yes** | **Yes** |
| Have all possible findings that could be found in the particular CBCT image are been reported? | **Yes** | **No** | **No** | **Yes** | **Yes** | **Yes** | **No** | **Yes** | **Yes** | **No** |
| Has an appropriately large sample been analyzed? | **No** | **Yes** | **Yes** | **Yes** | **Yes** | **Yes** | **Yes** | **No** | **No** | **No** |
| Has the prevalence of observed findings been assessed? | **No** | **Yes** | **Yes** | **Yes** | **Yes** | **Yes** | **Yes** | **Partly** | **No** | **No** |

**Supplementary Table 7.** Re-analysis of raw data of the Alsufyani 2017 study; percent of each incidentaloma among all patients with incidentalomas (27 patients).

| **Factor** | **Category** | **Statistic** |
| --- | --- | --- |
| Patient age | Mean (standard deviation) | 13.6 (3.1) |
|  | Range | 8.0, 18.0 |
|  |  |  |
| Patient gender | Female - n (%) | 16 (59%) |
|  | Male - n (%) | 11 (41%) |
|  |  |  |
| Incidental finding | Congenital; fusion - n (%) | 9/27 (33%) |
|  | Congenital; misalignment - n (%) | 1/27 (4%) |
|  | Congenital; ossicle - n (%) | 1/27 (4%) |
|  | Congenital; scoliosis - n (%) | 1/27 (4%) |
|  | Normal variant; vascular groove - n (%) | 5/27 (19%) |
|  | Normal variant; pharyngeal foveola - n (%) | 4/27 (15%) |
|  | Normal variant; ponticulus posticus - n (%) | 1/27 (4%) |
|  | Pathology - n (%) | 2/27 (7%) |
|  | Degenerative joint disease - n (%) | 1/27 (4%) |

**Supplementary Table 8.** Re-analysis of raw data of the Dogramaci 2014 study; percent of each incidentaloma among all patients with incidentalomas (107 patients / mean age 13.5, standard deviation 2.1 years / incidentalomas/patient median 3.0, interquartile range 2.0-4.0, range 1.0 to 9.0).

| **Category** | **Finding** | **Statistic** |
| --- | --- | --- |
| Airway | Maxillary sinus thickening - n (%) | 43/107 (40.2%) |
|  | Septum deviation - n (%) | 14/107 (13.1%) |
|  | Maxillary sinus thickening (both sides) - n (%) | 13/107 (12.1%) |
|  | Ossicle - n (%) | 5/107 (4.7%) |
|  | Nasal floor perforation - n (%) | 4/107 (3.7%) |
|  | Concha bullosa - n (%) | 3/107 (2.8%) |
|  | Polyp - n (%) | 1/107 (0.9%) |
|  |  |  |
| Bone | Thinning of cortical bone - n (%) | 27/107 (25.2%) |
|  | Bone dehiscence - n (%) | 1/107 (0.9%) |
|  | Bone sclerosis - n (%) | 1/107 (0.9%) |
|  |  |  |
| Jaw lesions | Dentigerous cyst - n (%) | 8/107 (7.5%) |
|  | Cyst - n (%) | 2/107 (1.9%) |
|  |  |  |
| Teeth | Enlarged follicle - n (%) | 61/107 (57.0%) |
|  | Root dilaceration - n (%) | 57/107 (53.3%) |
|  | Abnormal morphology - n (%) | 27/107 (25.2%) |
|  | Tooth impaction - n (%) | 21/107 (19.6%) |
|  | Caries - n (%) | 11/107 (10.3%) |
|  | Pulp calcification - n (%) | 11/107 (10.3%) |
|  | Root resorption - n (%) | 9/107 (8.4%) |
|  | Short root - n (%) | 8/107 (7.5%) |
|  | Periodontal ligament widening - n (%) | 7/107 (6.5%) |
|  | Retained root - n (%) | 7/107 (6.5%) |
|  | Dent invaginatus - n (%) | 4/107 (3.7%) |
|  | Ankylosis - n (%) | 3/107 (2.8%) |
|  | Odontoma - n (%) | 3/107 (2.8%) |
|  | Supernumerary teeth - n (%) | 3/107 (2.8%) |
|  | Enamel pearl - n (%) | 1/107 (0.9%) |
|  | Root fracture - n (%) | 1/107 (0.9%) |
|  | Tooth agenesis - n (%) | 1/107 (0.9%) |

_

**Supplementary Table 9.** Re-analysis of raw data of the Lopes 2016 study (27 patients with incidentalomas); summary of sample.

| **Factor** | **Category** | **Statistic** |
| --- | --- | --- |
| Patient age | Median (IQR) | 14.0 (11.0 to 15.0) |
|  | Range | 8.0 to 17.0 |
|  |  |  |
| Patient gender | Female - n (%) | 14/27 (52%) |
|  | Male - n (%) | 13/27 (48%) |
|  |  |  |
| Field of view | Maxilla - n (%) | 5/27 (19%) |
|  | Mandible - n (%) | 5/27 (19%) |
|  | Both jaws - n (%) | 17/27 (63%) |
|  |  |  |
| Voxel size | 0.20 - n (%) | 1/27 (4%) |
|  | 0.25 - n (%) | 13/27 (48%) |
|  | 0.30 - n (%) | 13/27 (48%) |
|  |  |  |
| Field of view size | 6 cm - n (%) | 10/27 (37%) |
|  | 13 cm - n (%) | 17/27 (63%) |
|  |  |  |
| X-ray reason | Orthodontics - n (%) | 12/27 (44%) |
|  | Cyst/tumor/fibro-osseous lesion - n (%) | 4/27 (15%) |
|  | Unerupted tooth - n (%) | 2/27 (7%) |
|  | 3^rd^ molar (and/or relation to nerve) - n (%) | 2/27 (7%) |
|  | Alveolar resorption - n (%) | 1/27 (4%) |
|  | Differential-diagnosis parotidal nodules - n (%) | 1/27 (4%) |
|  | Osteosynthesis material removal - n (%) | 1/27 (4%) |
|  | Root resorption - n (%) | 1/27 (4%) |
|  | Supernumerary localization - n (%) | 1/27 (4%) |
|  | Treatment planning; endodontics - n (%) | 1/27 (4%) |
|  | Treatment planning; implant - n (%) | 1/27 (4%) |
|  |  |  |
| Incidentaloma zone* | Airway - n (%) | 15/27 (56%) |
|  | Temporomandibular joint - n (%) | 16/27 (59%) |
|  | Bone - n (%) | 3/27 (11%) |
|  | Jaw lesion - n (%) | 1/27 (4%) |
|  | Teeth - n (%) | 18/27 (67%) |
|  | Soft-tissue calcification - n (%) | 6/27 (22%) |
|  |  |  |
| Total incidentalomas / zone | Airway - median (IQR) | 1.0 (0 to 2.0) |
|  | Temporomandibular joint - median (IQR) | 2.0 (0 to 2.0) |
|  | Bone - median (IQR) | 1.0 (-) |
|  | Jaw lesion - median (IQR) | 1.0 (-) |
|  | Teeth - median (IQR) | 2.0 (0 to 4.0) |
|  | Soft-tissue calcification - median (IQR) | 2.0 (2.0 to 3.0) |
|  |  |  |
| Incidentalomas / patient | Median (IQR) | 4.0 (2.0 to 7.0) |
|  | Range | 0 to 12.0 |

IQR, interquartile range

* with potential overlap among zones, as a patient might have incidental findings in more than one zone.

**Supplementary Table 10.** Re-analysis of raw data of the Lopes 2016 study; percent of each incidentaloma among all patients with incidentalomas (27 patients); individual findings.

| **Category** | **Finding** | **Statistic** |
| --- | --- | --- |
| Airway | Maxillary sinus thickening - n (%) | 11/27 (41%) |
|  | Maxillary sinus thickening (both sides) - n (%) | 6/27 (22%) |
|  | Cyst retention - n (%) | 3/27 (11%) |
|  | Turbinate hypertrophy - n (%) | 3/27 (11%) |
|  | Septum deviation - n (%) | 2/27 (7%) |
|  | Concha bullosa - n (%) | 1/27 (4%) |
|  | Maxillary sinus total coverage - n (%) | 1/27 (4%) |
|  |  |  |
| Temporomandibular joint | Osteophyte - n (%) | 11/27 (41%) |
|  | Flat condyle - n (%) | 9/27 (33%) |
|  | Flat condyle (both sides) - n (%) | 6/27 (22%) |
|  | Osteophyte (both sides) - n (%) | 6/27 (22%) |
|  | Missing zone - n (%) | 1/27 (4%) |
|  |  |  |
| Bone | Bone sclerosis - n (%) | 3/27 (11%) |
|  |  |  |
| Jaw lesions | Dentigerous cyst - n (%) | 1/27 (4%) |
|  |  |  |
| Teeth | Tooth impaction - n (%) | 13/27 (48%) |
|  | Impactions / patient - median (IQR) | 3.0 (2.0 to 4.0) |
|  | Tooth rotation - n (%) | 7/27 (26%) |
|  | Tooth rotations (multiple) - n (%) | 4/27 (15%) |
|  | Root dilaceration - n (%) | 4/27 (15%) |
|  | Root dilaceration (multiple) - n (%) | 3/27 (11%) |
|  | Enamel pearl - n (%) | 1/27 (4%) |
|  | Pulp calcification - n (%) | 1/27 (4%) |
|  | Supernumerary teeth - n (%) | 1/27 (4%) |
|  | Taurodontism - n (%) | 1/27 (4%) |
|  |  |  |
| Soft-tissue calcification | Style–hyoid complex calcification - n (%) | 6/27 (22%) |
|  | Style–hyoid complex calcification (both sides) - n (%) | 6/27 (22%) |
|  | Tonsilolith - n (%) | 1/27 (4%) |

IQR, interquartile range.

* with potential overlap among zones, as a patient might have incidentalomas in more than one zone.

**Supplementary Table 11.** Re-analysis of raw data of the Togan 2016 study; percent of each incidentaloma among all patients (48 patients) or only patients with incidentalomas (11 patients).

| **Factor** | **Category** | **Statistic** |
| --- | --- | --- |
| *All patients* |  |  |
| Patient age | Median (interquartile range) | 14.2 (11.7 to 16.2) |
|  | Range | 6.7 to 17.8 |
|  |  |  |
| Patient gender | Female - n (%) | 23/48 (48%) |
|  | Male - n (%) | 25/48 (52%) |
|  |  |  |
| Area | Cervical - n (%) | 11/48 (23%) |
|  | Sinus - n (%) | 14/48 (29%) |
|  | Both - n (%) | 23/48 (48%) |
|  |  |  |
| *Patients with incidentalomas* |  |  |
| Incidentaloma | Sinus finding - n (%) | 8/11 (73%) |
|  | Submandibular sialolithiasis - n (%) | 1/11 (9%) |
|  | Stylus-hyoid complex calcification - n (%) | 1/11 (9%) |
|  | Tonsilolith - n (%) | 1/11 (9%) |

_

**Supplementary Table 12.** Data from the Cobb 2013 study; reported at the level of findings among patients with incidentalomas (267 patients).

| **Location** | **Findings** | **Findings** | **% of findings** |
| --- | --- | --- | --- |
| Paranasal sinuses | Antral pseudocyst | 26 | 3.00% |
|  | Debris noted at max ostia | 2 | 0.23% |
|  | Hypoplastic antrum | 4 | 0.46% |
|  | Mucosal thickening (MT) of antrum | 29 | 3.34% |
|  | MT of antrum & ethmoid | 17 | 1.96% |
|  | MT of antrum, ethmoid, frontal | 2 | 0.23% |
|  | MT of atrum,ethmoid, sphenoid | 6 | 0.69% |
|  | MT of atrum, ethmoid, sphenoid, frontal | 2 | 0.23% |
|  | MT of atrum & sphenoid | 3 | 0.35% |
|  | MT of ethmoid | 21 | 2.42% |
|  | MT of ethmoid & frontal | 0 | 0.00% |
|  | MT of ethmoid & sphenoid | 2 | 0.23% |
|  | MT of frontal | 1 | 0.12% |
|  | MT of sphenoid | 4 | 0.46% |
|  | No sphenoid sinus | 1 | 0.12% |
|  | Obstructed mucocilliary pathway | 2 | 0.23% |
|  | Opacified antrum | 7 | 0.81% |
|  | Opacified frontal, ethmoid, and antrum | 1 | 0.12% |
|  | Opacified sphenoid sinus | 1 | 0.12% |
|  | Osteoma | 0 | 0.00% |
|  | Pneumatization of antrums | 0 | 0.00% |
|  | Pneumatization of sphenoid laterally | 34 | 3.92% |
|  | Severe rhinosinusitis | 1 | 0.12% |
|  | Sinus lift of left antrum | 0 | 0.00% |
|  | Soft tissue density in antrum | 3 | 0.35% |
|  | Soft tissue density in sphenoid | 5 | 0.58% |
| Nasal cavity | Cleft in nasal floor | 1 | 0.12% |
|  | Concha bullosa | 77 | 8.88% |
|  | Crenulated nasal septum | 0 | 0.00% |
|  | Deviated septum | 69 | 7.96% |
|  | Hypertrophy of turbinates | 3 | 0.35% |
|  | Nasal polyp | 4 | 0.46% |
|  | Rhinolith | 0 | 0.00% |
|  | Foreign body | 0 | 0.00% |
| Airway | Adenoid calcifications | 1 | 0.12% |
|  | Adenoid hypertrophy | 131 | 15.11% |
|  | Decreased airway due to calcified ligaments C1-C2 | 0 | 0.00% |
|  | Decreased airway due to patient positioning | 1 | 0.12% |
|  | Hypertrophy of nasopharyngeal tissues | 1 | 0.12% |
|  | Prevertebral thickening | 1 | 0.12% |
|  | Tonsillar calcifications | 3 | 0.35% |
|  | Tonsillar hypertrophy | 100 | 11.53% |
| Temporomandibular joint | Bifid condyle | 2 | 0.23% |
|  | Degen changes bilaterally | 2 | 0.23% |
|  | Degenerative left TMJ | 0 | 0.00% |
|  | Degenerative right TMJ | 1 | 0.12% |
|  | Hyperplastic unilateral condyle | 0 | 0.00% |
|  | Hypoplastic condyle bilaterally | 1 | 0.12% |
| Osseous structures | C1 posterior arch defect | 10 | 1.15% |
|  | C2 defect | 0 | 0.00% |
|  | Cleft palate | 2 | 0.23% |
|  | Degenerative changes in upper C-spine | 3 | 0.35% |
|  | Density changes in skull bones | 2 | 0.23% |
|  | Displacement of the dens due to trauma | 1 | 0.12% |
|  | Fusion of cervical vertebrae | 2 | 0.23% |
|  | High density in mandible | 23 | 2.65% |
|  | High density in maxilla | 14 | 1.61% |
|  | Lesion in mandible | 3 | 0.35% |
|  | Lesion in maxilla | 1 | 0.12% |
|  | Pneumatization of skull bones | 4 | 0.46% |
|  | Ponticulous ponticus | 2 | 0.23% |
|  | Soft tissue mass left zygomatic arch | 1 | 0.12% |
| Dental findings | Apical lesion | 5 | 0.58% |
|  | Apical resorption | 0 | 0.00% |
|  | Coronal fracture | 0 | 0.00% |
|  | Coronal lesion | 3 | 0.35% |
|  | Foreign debris in alveolar crest | 0 | 0.00% |
|  | Impacted teeth | 11 | 1.27% |
|  | Macrodont | 0 | 0.00% |
|  | Mesiodens | 3 | 0.35% |
|  | Microdont | 2 | 0.23% |
|  | Missing teeth | 20 | 2.31% |
|  | Retained permanent root | 1 | 0.12% |
|  | Retained primary tooth | 4 | 0.46% |
|  | Root fracture | 0 | 0.00% |
|  | Supernumerary | 3 | 0.35% |
|  | Transposition | 1 | 0.12% |
| Other | Asymmetric, large jugular foramen | 29 | 3.34% |
|  | Calcification in the area of the pineal gland | 26 | 3.00% |
|  | Calcification of petroclival ligaments | 3 | 0.35% |
|  | Calcification of stylomandibular ligaments | 2 | 0.23% |
|  | Calcifications of stylohyoid ligaments | 87 | 10.03% |
|  | Carotid artery calcifications | 0 | 0.00% |
|  | Debris in external auditory canal | 11 | 1.27% |
|  | Dural calcifications | 2 | 0.23% |
|  | Extracranial finding | 2 | 0.23% |
|  | Intracranial finding | 7 | 0.81% |
|  | Radiodense upper borders of thyroid cartilage | 0 | 0.00% |

**Supplementary Table 13.** Results of the Etamad 2023 study (250 patients).

| **Nr** | **Incidental finding category** | **Clinical significance** | **Absolute*** | **%** | **95% CI** |
| --- | --- | --- | --- | --- | --- |
| 1 | **Sino-nasal** |  | 112/250 | 44.80 | 38.53 to 51.19 |
| 2 | Nasal septal deviation | moderate | 135/250 | 54.00 | 47.61 to 60.30 |
| 3 | Concha bullosa | moderate | 44/250 | 17.60 | 13.09 to 22.90 |
| 4 | Haller cell | moderate | 11/250 | 4.40 | 2.22 to 7.74 |
| 5 | Frontal sinus | moderate | 98/250 | 39.20 | 33.11 to 45.55 |
| 6 | Mastoid cells over TMJ | moderate | 17/250 | 6.80 | 4.01 to 10.66 |
| 7 | Mucous retention cysts | moderate | 40/250 | 16.00 | 11.68 to 21.14 |
| 8 | Mucosal thickening of maxillary sinus | moderate | 119/250 | 47.60 | 41.27 to 53.99 |
| 9 | Accessory ostium | mild | 19/250 | 7.60 | 4.64 to 11.61 |
| 10 | Pansinusitis | severe | 4/250 | 1.60 | 0.44 to 4.05 |
| 11 | Mucosal thickening of ethmoid & sphenoid sinus | moderate | 5/250 | 2.00 | 0.65 to 4.61 |
| 12 | Ostia blockage | moderate | 3/250 | 1.20 | 0.25 to 3.47 |
| 13 | **Dentoalveolar** |  | 48/250 | 19.20 | 14.51 to 24.64 |
| 14 | Third molar | moderate | 108/250 | 43.20 | 36.97 to 49.59 |
| 15 | Supernumerary teeth | moderate | 11/250 | 4.40 | 2.22 to 7.74 |
| 16 | Missing teeth | moderate | 29/250 | 11.60 | 7.91 to 16.23 |
| 17 | Stafne's bone cavity | mild | 0/250 | 0.00 | 0.00 to 1.46 |
| 18 | Dilaceration | mild | 1/250 | 0.40 | 0.01 to 2.21 |
| 19 | Microdontia | mild | 2/250 | 0.80 | 0.10 to 2.86 |
| 20 | Root resorption | mild | 2/250 | 0.80 | 0.10 to 2.86 |
| 21 | Ectopic position | moderate | 3/250 | 1.20 | 0.25 to 3.47 |
| 22 | Torus mandibularis | mild | 1/250 | 0.40 | 0.01 to 2.21 |
| 23 | Odontogenic cyst | severe | 3/250 | 1.20 | 0.25 to 3.47 |
| 24 | Idiopathic osteosclerosis | moderate | 2/250 | 0.80 | 0.10 to 2.86 |
| 25 | Transposition | moderate | 3/250 | 1.20 | 0.25 to 3.47 |
| 26 | Pericoronitis | severe | 1/250 | 0.40 | 0.01 to 2.21 |
| 27 | **Naso-oropharyngeal airway** |  | 50/250 | 20.00 | 15.22 to 25.50 |
| 28 | Enlarged adenoids | moderate | 151/250 | 60.40 | 54.04 to 66.51 |
| 29 | Enlarged tonsils | mild | 47/250 | 18.80 | 14.15 to 24.20 |
| 30 | Tonsil calcification | mild | 17/250 | 6.80 | 4.01 to 10.66 |
| 31 | Vascular calcification | mild | 2/250 | 0.80 | 0.10 to 2.86 |
| 32 | **Temporomandibular joint** |  | 1/250 | 0.40 | 0.01 to 2.21 |
| 33 | Osteoarthritis | moderate | 3/250 | 1.20 | 0.25 to 3.47 |
| 34 | **Neck** |  | 1/250 | 0.40 | 0.01 to 2.21 |
| 35 | Cervical osteoarthritis | moderate | 2/250 | 0.80 | 0.10 to 2.86 |
| 36 | **Calcifications** |  | 36/250 | 14.40 | 10.29 to 19.37 |
| 37 | Stylohyoid ligament calcification | mild | 202/250 | 80.80 | 75.36 to 85.49 |
| 38 | Petroclinoid calcification | mild | 1/250 | 0.40 | 0.01 to 2.21 |
| 39 | Thyroid cartilage calcification | mild | 1/250 | 0.40 | 0.01 to 2.21 |
| 40 | Falx cerebri calcification | mild | 1/250 | 0.40 | 0.01 to 2.21 |
| 41 | **Miscellaneous findings** |  | 3/250 | 1.20 | 0.25 to 3.47 |
| 42 | Segmental maxillary odontodysplasia | moderate | 1/250 | 0.40 | 0.01 to 2.21 |
| 43 | Odontoma | mild | 2/250 | 0.80 | 0.10 to 2.86 |
